# Antibody-mediated clearance of an ER-resident aggregate that causes glaucoma

**DOI:** 10.1093/pnasnexus/pgae556

**Published:** 2024-12-10

**Authors:** Minh Thu Ma, Ahlam N Qerqez, Kamisha R Hill, Laura R Azouz, Hannah A Youngblood, Shannon E Hill, Yemo Ku, Donna M Peters, Jennifer A Maynard, Raquel L Lieberman

**Affiliations:** School of Chemistry & Biochemistry, Georgia Institute of Technology, 901 Atlantic Drive NW, Atlanta, GA 30332, USA; Department of Chemical Engineering, University of Texas at Austin, Austin, TX 78712, USA; School of Chemistry & Biochemistry, Georgia Institute of Technology, 901 Atlantic Drive NW, Atlanta, GA 30332, USA; Department of Chemical Engineering, University of Texas at Austin, Austin, TX 78712, USA; School of Chemistry & Biochemistry, Georgia Institute of Technology, 901 Atlantic Drive NW, Atlanta, GA 30332, USA; School of Chemistry & Biochemistry, Georgia Institute of Technology, 901 Atlantic Drive NW, Atlanta, GA 30332, USA; School of Chemistry & Biochemistry, Georgia Institute of Technology, 901 Atlantic Drive NW, Atlanta, GA 30332, USA; Department of Pathology & Laboratory Medicine, University of Wisconsin School of Medicine and Public Health, Madison, WI 53705, USA; Department of Ophthalmology & Visual Sciences, University of Wisconsin School of Medicine and Public Health, Madison, WI 53705, USA; Department of Chemical Engineering, University of Texas at Austin, Austin, TX 78712, USA; Department of Molecular Biosciences, University of Texas at Austin, Austin, TX 78712, USA; School of Chemistry & Biochemistry, Georgia Institute of Technology, 901 Atlantic Drive NW, Atlanta, GA 30332, USA

**Keywords:** myocilin, protein misfolding, autophagy, proteostasis, molecular recognition

## Abstract

Recombinant antibodies are a promising class of therapeutics to treat protein misfolding associated with neurodegenerative diseases, and several antibodies that inhibit aggregation are approved or in clinical trials to treat Alzheimer's disease. Here, we developed antibodies targeting the aggregation-prone β-propeller olfactomedin (OLF) domain of myocilin, variants of which comprise the strongest genetic link to glaucoma and cause early onset vision loss for several million individuals worldwide. Mutant myocilin aggregates intracellularly in the endoplasmic reticulum (ER). Subsequent ER stress causes cytotoxicity that hastens dysregulation of intraocular pressure, the primary risk factor for most forms of glaucoma. Our antibody discovery campaign yielded two recombinant antibodies: anti-OLF1 recognizes a linear epitope, while anti-OLF2 is selective for natively folded OLF and inhibits aggregation in vitro. By binding OLF, these antibodies engage autophagy/lysosomal degradation to promote degradation of two pathogenic mutant myocilins. This work demonstrates the potential for therapeutic antibodies to disrupt ER-localized protein aggregates by altering the fate of folding intermediates. This approach could be translated as a precision medicine to treat myocilin-associated glaucoma with in situ antibody expression. More generally, the study supports the approach of enhancing lysosomal degradation to treat proteostasis decline in glaucoma and other diseases.

**Significance Statement**

Primary open angle glaucoma is the leading form of glaucoma, causing retinal degeneration and irreversible vision loss in many millions of individuals worldwide. Nonsynonymous mutations in the gene encoding myocilin cause early onset familial glaucoma. Inspired by the development of antibodies for other age-onset neurodegenerative diseases, here we developed new antibodies that target the domain of myocilin that misfolds in the endoplasmic reticulum (ER) and causes pathogenic cytotoxicity. We show that these antibodies degrade aggregating mutant myocilin in situ by rerouting mutant myocilin for lysosomal degradation. Our study represents a proof of concept that antibodies can degrade proteins retained in the ER. More generally, addressing proteostasis decline in anterior eye tissues holds promise to combat the development of glaucoma.

## Background

Recombinant antibodies represent an emerging class of versatile and powerful therapeutics to treat protein conformational and misfolding diseases (1). Several antibodies capable of inhibiting aggregation are approved or in clinical trials to treat amyloidoses and neurodegenerative conditions such as Alzheimer's, Parkinson's, and prion diseases (2, 3). The therapeutic potential of antibodies is derived from their ability to recognize specific epitopes on the target antigen that may only be exposed during certain stages of the protein life cycle. This feature provides opportunities to neutralize the pathological aggregates of misfolded proteins: antibodies can block aggregation by stabilizing the folded state of a protein, targeting aggregates for cellular degradation, or disrupting aggregate formation (4, 5).

Glaucoma is a heterogeneous group of neurodegenerative ocular diseases characterized by gradual and irreversible damage to the optic nerve that affects more than 70 million individuals worldwide (6). In end stage disease, glaucoma patients often suffer permanent vision loss. Indeed, glaucoma is the second leading cause of blindness. The quintessential glaucoma risk factor is elevated intraocular pressure (IOP), which is regulated by the balanced production and drainage of aqueous humor, a clear fluid that nourishes the ocular anterior segment. In the healthy eye, aqueous humor is continuously drained through the mechanosensitive trabecular meshwork (TM) tissue located between the sclera and cornea. Conversely, in most forms of glaucoma, the TM is diseased and TM cellularity is diminished, leading to impaired drainage of aqueous humor and increased IOP (7–9).

**Fig. 1.**
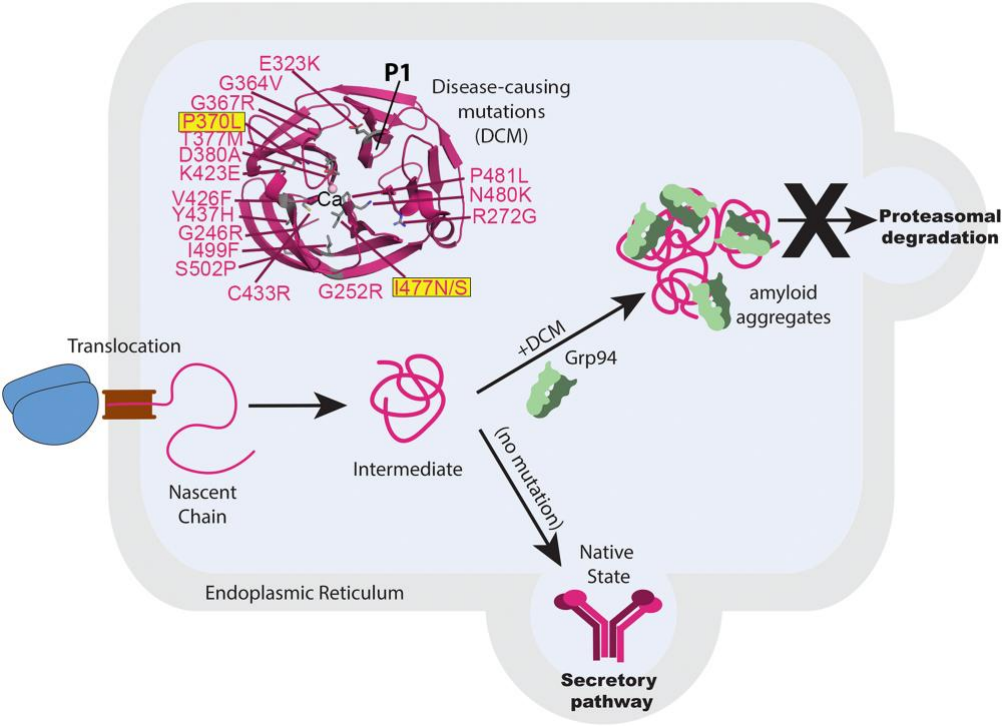
Overview of pathogenic mechanism underlying myocilin-associated glaucoma. Myocilin is a secreted glycoprotein that is folded in the ER. Individual rare disease-causing mutations within the myocilin OLF domain (PDB: 4WXQ), including P370L and I477N studied here (boxed, highlighted), lead to mutant myocilin sequestration in the ER and misfolding. Mutant myocilins accumulate due to an aberrant interaction with Grp94 that prevents retro-translocation for proteasomal degradation. Mutant myocilin aggregates exhibit numerous hallmarks of amyloid. P1 is the amyloid peptide stretch proposed to template amyloid formation (see text).

The protein myocilin is secreted at a relatively high level to the TM extracellular matrix. Myocilin missense mutations comprise the strongest genetic link to open angle glaucoma (OAG), estimated to account for more than 3 million cases of heritable primary open angle glaucoma and more than 150,000 cases of juvenile-onset open angle glaucoma (10–14). The C-terminal olfactomedin (OLF) domain of myocilin houses ∼90% of disease-causing mutations (Fig. 1), making the heritable subtype of glaucoma attractive for precision medicine and disease-modifying therapeutics (12, 15). The pathogenesis of myocilin-associated glaucoma is associated with a toxic gain of function (16). Specifically, disease-causing mutations within OLF lead to mutant myocilin aggregation and accumulation within the endoplasmic reticulum (ER) (17–19) (Fig. 1). Although misfolded proteins in the ER are typically subjected to proteasomal degradation by ER chaperone machinery, clearance of OLF-resident myocilin mutants is hindered by aberrant interactions with molecular chaperones such as glucose regulated protein 94 (Grp94), leading to the accumulation of misfolded proteins (Fig. 1), ER stress, and subsequent TM cell death (20–23). This molecular cascade culminates in an accelerated timeline for glaucoma-associated ocular hypertension.

Despite decades of effort, the only method for slowing optic nerve damage and delaying vision loss caused by all types of glaucoma is reducing IOP pharmacologically or surgically (24–26). Whereas antibody therapies are widely available for other ocular conditions, notably Lucentis (ranibizumab injection) to treat age-related wet macular degeneration (27, 28), no such treatment yet exists for glaucoma. Here, we report the first OLF-targeting antibodies that enable degradation of aggressive mutants through cellular mechanisms afforded by molecular recognition for the antibody with OLF. In situ expression of these antibodies in the ER, e.g. by gene therapy (29) or potentially other delivery mechanisms, holds promise as a new approach to treat the underlying cause of glaucoma and illustrates a new therapeutic modality for other protein misfolding diseases.

## Results

### Recombinant antibody discovery and initial characterization

BALB/C mice were immunized with purified human OLF, boosted with mouse OLF and boosted again with a 1:1 mixture of human and mouse OLF, an approach that resulted in serum reactivity against both human and mouse OLF (Fig. S1A). The antibody variable regions were then amplified from mouse spleen mRNA and used to generate a phage scFv library with 10^9^ independent clones. Panning against anti-c-myc selected for phage expressing full-length scFvs was followed by panning on human OLF and then two rounds against either human or mouse OLF (Fig. S1B). Individual clones from all rounds, 77 in total, were analyzed by Sanger sequencing and the 21 unique clones analyzed for binding to mouse and human OLF via phage ELISA (Fig. S1C and D). Six scFv sequences were selected for further analyses due to their unique complementarity determining regions (CDRs) and OLF binding profiles that included strong binding to human OLF or strong cross-reactive binding to human and mouse OLF (Fig. S1D and E).

**Fig. 2.**
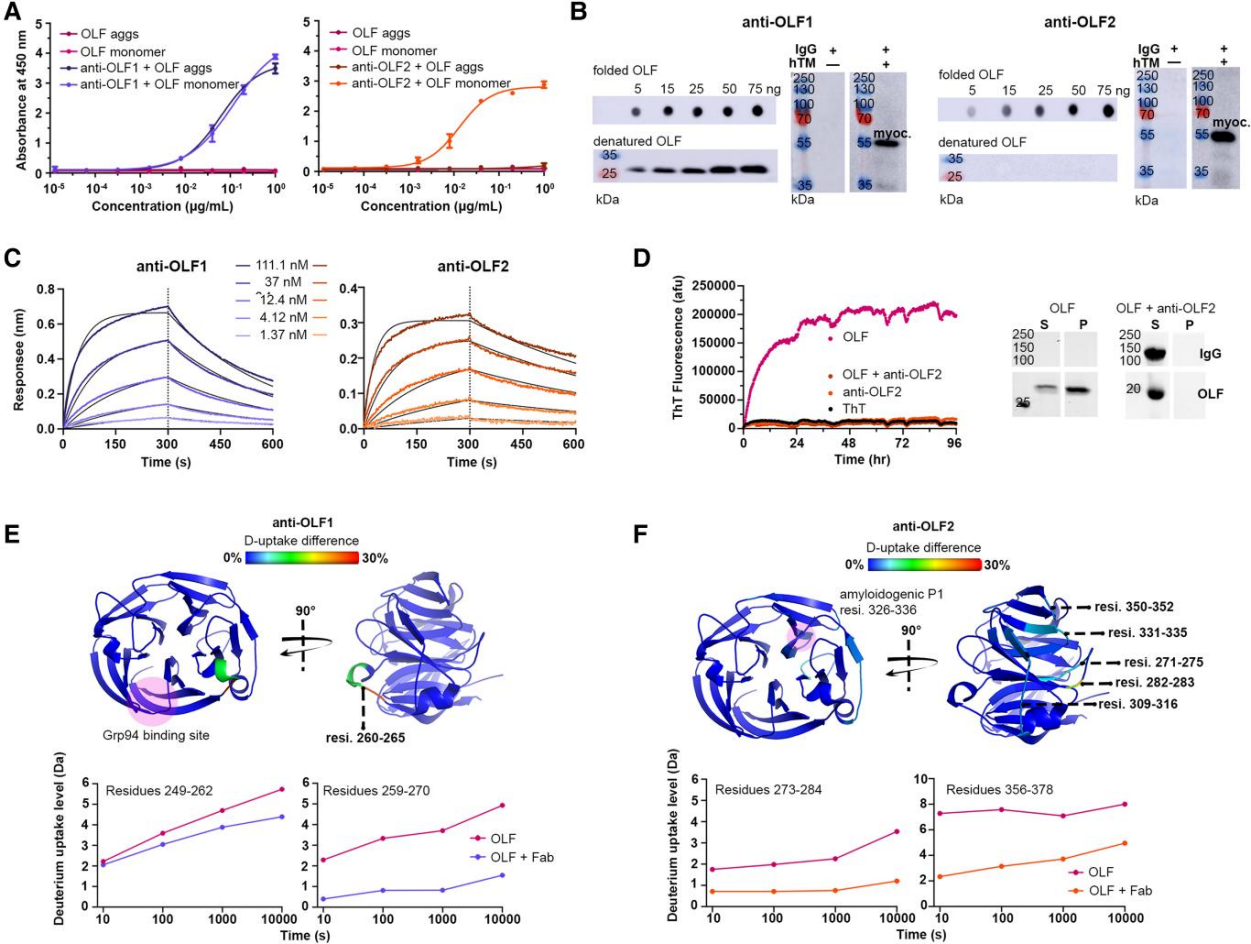
Anti-OLF1 and anti-OLF2 antibodies exhibit unique conformational selectivity and aggregation inhibition properties in accordance with their binding modes. A) ELISA against folded, monomeric WT OLF and soluble amyloidogenic aggregates isolated after size exclusion chromatography (OLF aggs, see Methods) showing nonselectivity of anti-OLF1 (left) and selectivity of anti-OLF2 for monomeric OLF (right). Data are representative of at least two independent experiments each with three analytical replicates. B) Conformational specificity of anti-OLF1 (left) and anti-OLF2 (right). Dot blot (folded OLF, top) and Western blot (denatured OLF, bottom) against nanogram quantities of purified OLF. Immunoprecipitation with buffer or spent hTM media followed by Western blot reveals isolation of endogenous full-length myocilin (∼55 kDa) by anti-OLF1 and anti-OLF2. Data represent at least two independent preparations of the target antigens. C) Binding of anti-OLF1 and anti-OLF2 Fab to human OLF measured by bio-layer interferometry indicates high binding affinity for both antibodies. *k*_on_ and *k*_off_ rates were determined by fitting collected data into a 1:1 Langmuir binding model. *K_D_* values were calculated from *k*_on_/*k*_off_ and SD were determined from globally fitting all concentrations. Data collected are showed as colored lines and fits as black lines, representing two independent experiments; see also Table S2. D) Thioflavin-T fluorescence aggregation assay (left) reveals anti-OLF2 inhibits aggregation of purified WT OLF at 1:1 molar ratio. SDS-PAGE analysis (right) of soluble fraction (S) and pellet (P) at the conclusion of the assay confirms rescue of OLF aggregation by anti-OLF2. Data are representative of two independent experiments. Full blots and gels in (B) and (D) are presented in Fig. S2A–C. E, F) Deuterium uptake difference between unbound and antibody-bound WT OLF elucidated by HDX-MS after 10,000 s of deuterium exchange is projected onto the structure of WT human myocilin OLF (PDB: 4WXQ). The difference in deuteration is showed in range from 0% (left, blue) to 30% (right, red). The OLF residues participating in the binding epitopes of anti-OLF1 Fab (E) or anti-OLF2 Fab (F). Shaded sphere (pink) indicates previously identified location of the Grp94 binding site (E) or the aggregation-prone peptide P1 (F). Deuterium uptake level at each labeling time point for selected epitope-participating OLF residues are plotted below the structures. See also Figs. S5–S8.

We first evaluated the extent to which antibodies recognize epitopes specific to native or misfolded myocilin. The selected scFv clones were converted to chimeric antibodies comprising the murine variable domains with human IgG1/kappa constant domains and interrogated for the ability to detect monomeric, aggregated, or unfolded OLF protein using Western blot, dot blot, and ELISA (Figs. 2A and B, S2A and B, and S3A–D). Antibodies recognizing linear epitopes are expected to detect monomeric OLF and aggregates obtained during purification (see Methods) in Western blot and ELISA. By contrast, antibodies recognizing conformational epitopes are expected to preferentially detect native/monomeric OLF in dot blot and ELISA, which does not perturb protein conformation. Of these six IgGs, two unique antibodies emerged. Anti-OLF1 (initially called OLF-41, Fig. S1, Table S1) detected folded, unfolded, and misfolded OLF samples in all formats indicating a linear epitope. Anti-OLF2 (initially called OLF-46, Fig. S1, Table S1) detected only folded OLF in dot blot and ELISA, denoting recognition of a conformational epitope (Fig. 2A and B). Both antibodies immunoprecipitated full-length myocilin from conditioned cell media of primary human TM cells from patient donors (Fig. 2B), demonstrating robust binding to the OLF domain in the context of the full-length protein.

The binding properties of anti-OLF1 and anti-OLF2 were evaluated further after proteolyzing the IgGs to monomeric Fab fragments (see Methods) and biotinylating human OLF (Fig. S3E). Bio-layer interferometry measurements confirmed low nanomolar binding affinities to human OLF for both anti-OLF1 and anti-OLF2 (Fig. 2C, Table S2). No binding was detected when anti-OLF1 or anti-OLF2 was incubated with a panel of reagents possessing diverse biochemical features, suggesting a high degree of antigen specificity and lack of polyspecificity (Fig. S3F). In sum, antibodies anti-OLF1 and anti-OLF2 exhibit strong biophysical properties across all experiments, with the principal difference being their OLF binding mode. Notably, the sequences of anti-OLF1 and anti-OLF2 were each represented just once among the panel of 77 antibodies characterized, indicating these characteristics are uncommon (Fig. S1E).

### Conformational antibody anti-OLF2 rescues aggregation of OLF in vitro

Motivated by the observation that anti-OLF2 binds a folded epitope, we examined the effects of antibody binding on the aggregation of wild-type (WT) human OLF in vitro. In our established assay, purified WT OLF is driven to fibrillize with slightly elevated temperature (42 °C), which is detected by fluorescence of the amyloid dye Thioflavin-T (ThT) (30, 31). Whereas a strong increase in aggregation is observed when WT OLF is incubated alone, the addition of anti-OLF2 but not anti-OLF1 inhibits WT OLF aggregation at 42 °C, observed as a baseline level of ThT fluorescence throughout the 96 h assay. Both the IgG and Fab formats of anti-OLF2 inhibited aggregation (Figs. 2D, S2C, and S4A). At molar ratio of 1 OLF to 1 bivalent IgG molecule with two antigen binding sites, OLF aggregation is completely abrogated. At a molar ratio of 2 OLF to 1 IgG or Fab, of which the latter contains just one antigen binding site, aggregation is diminished to a large extent (Fig. S4A). Remarkably, instead of forming an insoluble fibrillar material, both WT OLF and anti-OLF2 remain in solution at the conclusion of the assay (Fig. 2D) indicating that OLF aggregation was inhibited in the presence of anti-OLF2. By contrast, the presence of anti-OLF1 accelerated WT OLF aggregation in this assay (Fig. S4B), likely due to its nonselectivity for folded OLF. In support of the conclusion that this effect is antibody specific, an IgG triaged earlier in the process (anti-OLF4) had no effect on OLF aggregation in the assay (Fig. S4C).

### In accordance with their binding mode, anti-OLF1, and anti-OLF2 engage different epitopes on OLF

Consistent with our data suggesting anti-OLF1 binds a linear epitope with no conformational selectivity, hydrogen-deuterium exchange mass spectrometry (HDX-MS, see Methods) revealed a difference in relative deuterium uptake between unbound and antibody-bound OLF on a surface exposed loop spanning residues 260–265 (green to orange region, Figs. 2E, S5A, and S6). This loop is in proximity of the previously identified binding site for aberrant binding of Grp94, the ER-resident heat shock protein 90 paralog that recognizes mutant myocilin but, counterintuitively, prevents proteasomal clearance (32), by co-aggregating with mutant myocilin (33, 34).

Consistent with dot blot and Western blot data suggesting anti-OLF2 binds a conformational OLF epitope, HDX-MS analysis revealed a discontinuous epitope, including residues 271–275, 282–283, 309–316, 331–335, and 350–352 (light blue to yellow regions, Figs. 2F, S5B, S7, and S8), on a different propeller blade and remote from the linear binding site identified for anti-OLF1. The binding site of anti-OLF2 is adjacent to one of two experimentally validated amyloid-prone peptide regions (APR) of OLF known as P1 that are thought to form the amyloid core of myocilin aggregates (30, 35, 36).

### Anti-OLF1 and anti-OLF2 alter the localization of intracellular myocilin expressed in an immortalized TM cell line

**Fig. 3.**
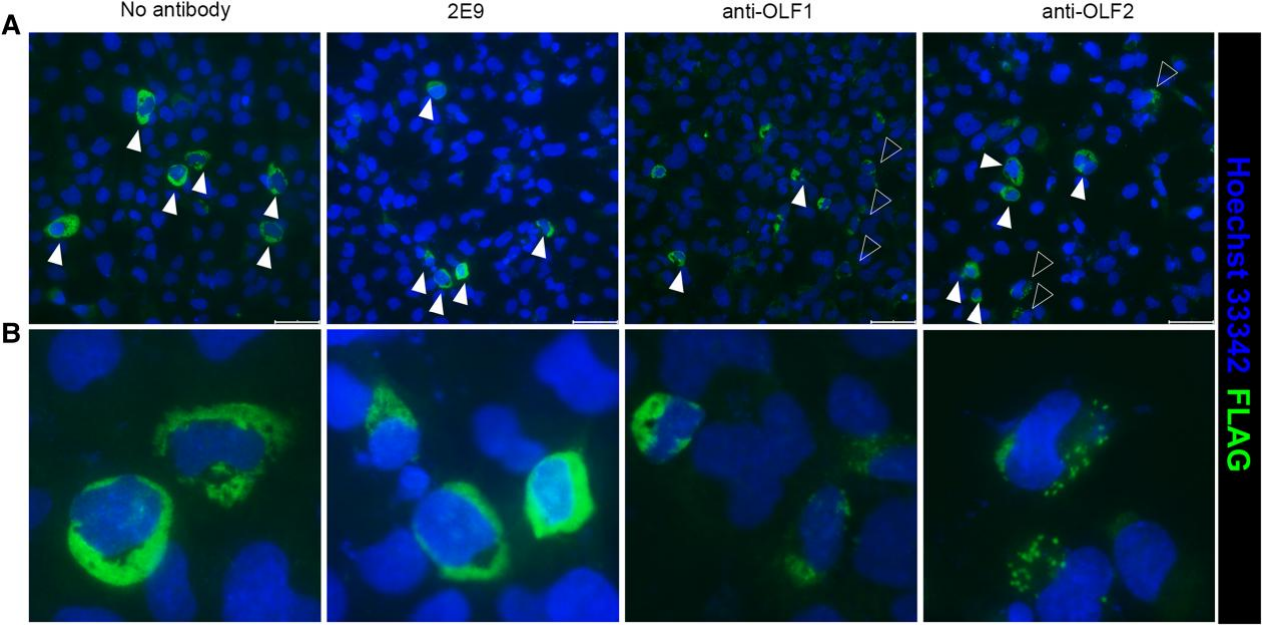
Co-expression of myocilin^P370L^ with anti-OLF1 or anti-OLF2 alters intracellular localization of myocilin^P370L^ in HTM-1 cells. A) Top, immunofluorescence images corresponding to myocilin^P370L^ expression alone, with control antibody 2E9, anti-OLF1 or anti-OLF2. Filled triangle, cells with perinuclear/ER localization. Open triangles, cells with lower intensity puncta consistent with lysosomes. Scale bar = 50 μm. B) Zoom in view of a portion of panels in A. HTM-1 cells were stained with an Alexa Fluor 488-conjugated anti-FLAG (L5) antibody. Images are representative of two independent experiments. See also Fig. S9.

We next assayed the effect of anti-OLF1 and anti-OLF2 on cellular trafficking of a severe glaucoma-causing myocilin variant, P370L (37). Patient-derived primary human TM cells (HTM) harboring mutant myocilins are not available for research and primary HTM cell lines cultured in the lab are generally recalcitrant to manipulation, becoming senescent after ∼6 passages and exhibiting poor transfection efficiency for routine cell culture experiments and analyses (38). Accordingly, we identified experimental conditions to test the effect of co-transfection of myocilin with antibodies in an immortalized HTM cell line, HTM-1, which retains most characteristics of primary HTM cells but can be cultured for additional passages (39). We co-transfected HTM-1 cells with the target gene (myocilin^P370L^ or green fluorescence protein (GFP) control) alone or with antibody plasmids at levels that minimized cell death. Transfection efficiency is in line with expectations based on plasmid levels used for transfection and literature precedent (39), and transfected cells are visible by immunofluorescence staining (Figs. 3 and S9). Antibody expression did not affect the localization of expressed GFP (Fig. S9). There was also no discernable effect of control antibody 2E9 on localization of GFP or myocilin^P370L^ (Fig. 3). Interestingly, the presence of anti-OLF1 or anti-OLF2 changes the localization of mutant myocilin^P370L^ from the expected perinuclear bands of the ER (40) to scattered puncta (Fig. 3). Thus, anti-OLF1 and anti-OLF2 appear to redirect myocilin^P370L^ to a different subcellular location in physiologically relevant HTM-1 cells.

### Anti-OLF1 and anti-OLF2 promote degradation of full-length mutant myocilin in a HEK293T model cellular secretion assay

Given the limitations of TM cells, we turned to two robust and manipulatable HEK293T-based cell models to further analyze the fate of mutant myocilins in the presence of anti-OLF1 or anti-OLF2. In the first model, HEK293T cells are transiently transfected with a plasmid encoding the myocilin of interest, e.g. myocilin^P370L^ or myocilin^WT^ (HEK^P370L^, HEK^WT^). The second model is an HEK293T inducible cell line in which expression of myocilin^I477N^ or myocilin^WT^ is induced with doxycycline (iHEK^I477N^, iHEK^WT^) (41, 42). In both cell models, myocilin is tracked in the (i) cell growth media after secretion, (ii) intracellular Triton X-100 soluble fraction, and (iii) intracellular Triton X-100 insoluble fraction. Myocilin^P370L^ and myocilin^I477N^ are pathogenic variants established to be sequestered intracellularly in an insoluble fraction (43).

**Fig. 4.**
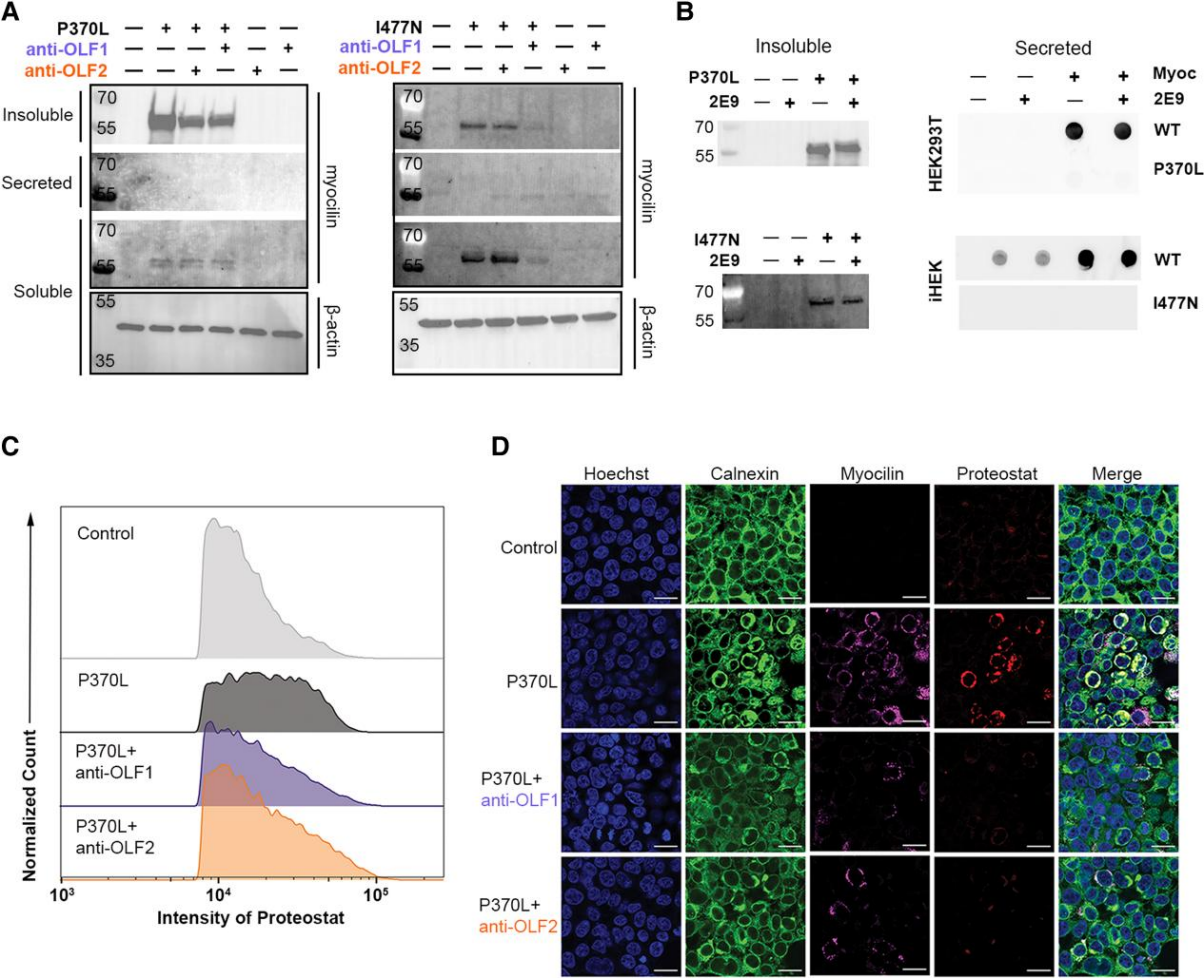
Anti-OLF1 and anti-OLF2, but not control antibody 2E9, inhibit aggregation and promote degradation of disease-causing myocilin variants in two HEK293T cellular models. A) Western blot analysis of cellular secretion assay fractions demonstrating the effects of anti-OLF1 and anti-OLF2 on detergent-insoluble myocilin lysates (top blot), secreted myocilin from spent cellular media (middle blot), and soluble myocilin (bottom blots) from HEK293T cells transfected with myocilin^P370L^ (left) and induced iHEK^I477N^ cells (right). Level of soluble β-actin was used as a loading control for the soluble fraction. B) Levels of insoluble (left) or secreted (right) myocilin^P370L^ or myocilin^I477N^ do not change as a result of the presence or absence of control antibody 2E9. C) Representative flow cytometry results for HEK293T cells transfected with myocilin^P370L^ and either anti-OLF1 or anti-OLF2 and stained with PROTEOSTAT. D) Representative immunofluorescence images and colocalization analysis of HEK293T cells transfected with myocilin^P370L^ and stained with PROTEOSTAT. Full blots, quantification, and statistical significance are presented in Fig. S10.

Co-expression of myocilin^P370L^ or myocilin^I477N^ with either anti-OLF1 or anti-OLF2 leads to degradation of mutant myocilin (Figs. 4A and S10). In the absence of antibody, myocilin^I477N^ is not secreted, rather, it is detected to a limited extent in the soluble fraction and prominently in insoluble cellular fractions, as expected. Myocilin^P370L^ is also not secreted, with a very weak soluble band and prominent insoluble band in Western blot. When anti-OLF1 or anti-OLF2 is present in either cell line, there is less insoluble mutant myocilin (Figs. 4A and S10A–C and F). In the case of myocilin^I477N^, a decrease in the soluble fraction is observed, which is particularly prominent for anti-OLF1.

### The effects of anti-OLF1 and anti-OLF2 are specific to mutant myocilin misfolding

Three sets of control experiments support the conclusion that anti-OLF1 and anti-OLF2 effects are specific to mutant myocilin misfolding and not a general effect of antibody/mutant myocilin co-expression in HEK293T cells. First, neither the presence of anti-OLF1 nor anti-OLF2 affects myocilin^WT^ secretion in either cell model (Fig. S10D), indicating neither antibody promotes sequestration of myocilin^WT^ intracellularly. Second, transfection with a control antibody, 2E9, does not decrease the insoluble fraction or alter secretion of myocilin^WT^, myocilin^I477N^, or myocilin^P370L^ (Figs. 4B and S10E and F). In fact, quantification reveals that 2E9 increases the insoluble myocilin fraction for myocilin^I477N^, the opposite effect observed with anti-OLF1 and anti-OLF2 (Fig. S10E and F). Third, the intracellular localization of anti-OLF1 and anti-OLF2 tracks with mutant myocilin localization (Fig. S11). When anti-OLF1 or anti-OLF2 are co-expressed with myocilin^I477N^ or myocilin^P370L^, antibody levels in the soluble fraction decrease while levels in the insoluble fraction increase (Fig. S11A and B). By contrast, intracellular localization of the control antibody 2E9 is unaffected by expression of myocilin^I477N^ in the iHEK^I477N^ model (Fig. S11C). In HEK^P370L^ involving transient co-expression of 2E9 and myocilin^P370L^, levels of insoluble 2E9 are unchanged compared to levels of 2E9 without co-expression of myocilin^P370L^, and levels of soluble 2E9 are lower (Fig. S11D). The difference in effect of 2E9 for the transient versus stable cell lines is likely the difference in the method used to express mutant myocilin because co-expression of 2E9 and myocilin^WT^ in iHEK^WT^ also yields somewhat lower soluble levels of antibody compared to control lacking myocilin^WT^ (Fig. S11E). Taken together, the reduction in insoluble mutant myocilin is not an artifact of co-expressing antibodies with mutant myocilin in the ER, but rather specific to the interaction between anti-OLF1 or anti-OLF2 and their misfolding molecular target.

### Anti-OLF1 and anti-OLF2 reduce intracellular aggregates in HEK293T transfected with myocilin^P370L^

Additional evidence for reduced insoluble intracellular mutant myocilin levels when co-expressed with anti-OLF1 or anti-OLF2 were obtained by analysis of amyloid staining with PROTEOSTAT by flow cytometry and immunofluorescence. In flow cytometry, cells transfected with myocilin^P370L^ alone display a high level of PROTEOSTAT signal, indicating the presence of aggregates. Co-transfection of myocilin^P370L^ with either anti-OLF1 or anti-OLF2 reverts the PROTEOSTAT signal near to levels observed with nontransfected cells (Figs. 4C, S10F, and S12). The aggregation propensity factor (APF) increases significantly in cells transfected with myocilin^P370L^ as compared to nontransfected control, whereas in the presence of anti-OLF1 or anti-OLF2, the APF significantly decreases (Figs. 4C and S10F). Similarly, immunofluorescence imaging confirms that myocilin^P370L^ is sequestered intracellularly, co-localizes with the ER marker calnexin and is positive for the amyloid dye PROTEOSTAT (Figs. 4D and S10F). Consistent with results from flow cytometry, PROTEOSTAT intensity decreases significantly when myocilin^P370L^ is co-transfected with either anti-OLF1 or anti-OLF2 (Figs. 4D and S10F). In sum, co-expression of anti-OLF1 or anti-OLF2 reduces the accumulation of intracellular myocilin^P370L^ amyloid aggregates sequestered in the ER.

### Anti-OLF1 and anti-OLF2 enable clearance of mutant myocilin by promoting lysosomal degradation

**Fig. 5.**
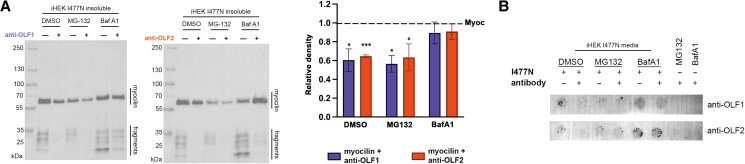
Anti-OLF1 and anti-OLF2 degrade mutant myocilin through an apparent lysosomal pathway. A) Western blot analysis of the detergent-insoluble fraction from induced iHEK^I477N^ cells treated with DMSO, the proteasomal inhibitor MG-132, or the lysosomal inhibitor Baf A1 for 24 h following transient transfection of anti-OLF1 (left) or anti-OLF2 (middle). Quantification of Western blots of insoluble myocilin^I477N^ from two independent experiments, with SD compared to myocilin level in the absence of antibody within each treatment. *P < 0.05; ***P < 0.0001 relative density compared to myocilin^I477N^ alone. B) Dot blot analysis of spent media fraction shows extremely low levels of secreted myocilin^I477N^ across these experiments.

To evaluate the route of mutant myocilin degradation, we employed the iHEK^I477N^ cell line, which is thought to be overall more comparable to TM cells than the transient overexpression model because it is prolonged expression of lower levels of mutated myocilin that is deleterious to cells (41). A plasmid encoding for anti-OLF1 or anti-OLF2 was transfected into iHEK^I477N^; 24 h later, myocilin^I477N^ expression was induced with doxycycline, and 24 h later, cells were treated with MG-132 (44) or Bafilomycin A1 (Baf A1) (45) to inhibit proteosomal or autophagy–lysosomal degradation, respectively. Treatment with MG-132 resulted in no change to antibody-mediated degradation of myocilin^I477N^ (Fig. 5A). In contrast, treatment with Baf A1 blocked antibody-mediated degradation and instead increased the level of intracellular aggregates (Fig. 5A). There was no perceptible change in secreted myocilin^I477N^ levels above baseline with any of the treatments (Fig. 5B). Thus, in line with previous studies indicating that proteasomal degradation is impaired in cells expressing mutant myocilin (20), and the change observed upon co-transfection of myocilin^P370L^ and antibodies in HTM-1 cells, anti-OLF1 and anti-OLF2-mediated mutant myocilin degradation likely involves the autophagy–lysosomal pathway.

## Discussion

Inspired by recent advances in antibody-based therapeutics to treat protein conformational neurodegenerative diseases (46–49), we hypothesized that antibodies could thwart mutant myocilin misfolding and the subsequent accumulation in the ER that confers a toxic gain-of-function and downstream pathology. The TM eye tissue is continually subjected to mechanical, oxidative, and phagocytotic stressors. Accordingly, TM cells are equipped to handle these insults with robust protective and repair mechanisms (50). Despite these features, the introduction of numerous different single nonsynonymous point mutations in the myocilin OLF domain leads to TM cell death and an accelerated time frame for IOP elevation and glaucoma-associated vision loss (51).

Unlike other misfolding diseases in which pathogenic aggregation originates from an intrinsically disordered protein found in the extracellular space where it is accessible to circulating antibodies and mechanisms that remove small immune complexes (52, 53), OLF-mediated aggregation of mutant myocilin forms intracellularly during folding to its native state in the ER. Antibodies targeting mutant myocilin, either to inhibit aggregation or enable degradation of aggregated species, need to act in a complex, crowded, and dynamic protein folding environment. Such antibodies themselves will also interface with intracellular protein quality control processes like chaperones. Thus, strategies to ameliorate the underlying pathogenesis of myocilin-associated glaucoma with antibodies poses challenges not present in better studied misfolding systems.

In contrast to antibodies targeting amyloid-β (54, 55) or tau (56) for the treatment of Alzheimer disease (57) or α-synuclein for the treatment of Parkinson's disease (58, 59), targeting a specific misfolded myocilin species may not be required to promote mutant myocilin degradation. Our antibody discovery campaign yielded two high-affinity antibodies targeting different OLF surface epitopes: anti-OLF1 recognizes an exposed loop that allows it to bind multiple OLF conformational states, whereas anti-OLF2 recognizes a discontinuous epitope near the proposed APR P1 (30) that restricts binding to native OLF conformations. Interestingly, antibodies binding conformational epitopes were not widely represented among selected clones (Fig. S3A–D). Because anti-OLF1 accelerated OLF aggregation in vitro, initially we presumed that the co-expression of anti-OLF1 with mutant myocilin would exacerbate the mutant myocilin aggregation phenotype. Conversely, we were optimistic that because anti-OLF2 abrogated aggregation in vitro, co-expression of anti-OLF2 with mutant myocilin would stabilize the native OLF conformation against the partial unfolding event that necessarily precedes its fibrillization (30). The mechanism we initially envisioned for anti-OLF2 was analogous to how small-molecule stabilizers of the cystic fibrosis conductance regulator (60) or transthyretin (61, 62) correct trafficking or aggregation, respectively, strategies that have been successfully translated to the clinic. Specifically, we anticipated that anti-OLF2 would obfuscate mutant myocilin from detection by the proteostasis network and rescue myocilin secretion. In turn, extracellular mutant myocilin degradation would be handled via the robust phagocytosis activities of TM cells (63). Instead, both anti-OLF1 and anti-OLF2, but not the control antibody 2E9, promoted mutant myocilin clearance. Experimental evidence indicates engagement of the lysosomal degradation pathway, not proteasomal degradation, of which the latter might have involved a TRIM21-dependent mechanism (64). Future studies will be aimed at probing the mechanism of antibody-mediated degradation of mutant myocilin in additional molecular detail, including whether the underlying cellular mechanism for each anti-OLF antibody is the same or different.

Our antibodies expand the applicability of high-affinity therapeutics to target and degrade misfolded proteins in the ER. The approach does not require the use of known ligands for the target, overcoming challenges posed by small-molecule degradation technologies such as proteolysis targeting chimeras (65, 66) and related platforms (67–69). Expression of antibodies in the ER is distinct from antibody-based degradation technologies that exploit the versatility of antibodies to degrade membrane-associated proteins via recruiting E3 ligases (AbTACs (70) and PROTABs (71)) or by utilizing lysosomal-targeting receptors (LYTACs (72)). Finally, in contrast to intrabodies (73), antibody fragments in development for cytosolic targets including several prone to misfolding, our ER-targeted nucleic acid-delivered antibodies can be expressed in their full length with native disulfide bonds, do not require engineering for stability (74), or additional functionalization for effective targeting to the proteasome (75) or lysosome (75).

Current management of myocilin-associated glaucoma in the clinic is the same as age-onset OAG, which involves lowering IOP. However, myocilin-associated glaucoma is one of the strongest examples of autosomal dominant inherited Mendelian disease (11, 76, 77), and recent developments in differentiating characteristics of disease variants from benign polymorphisms make myocilin an attractive candidate for precision medicine that targets the underlying disease process. To that end, to date, therapeutic development for myocilin-associated glaucoma has focused on manipulating the ER stress signaling pathway, CRISPR, and Grp94. For example, we have shown that the interaction between Grp94 and mutant myocilin can be abrogated with selective small molecules targeting Grp94 or by downregulation of Grp94 with siRNA (20, 78). Treatment with Grp94-targeted small molecules or siRNA leads to clearance of mutant myocilin by autophagy (20, 78), similar to what we observed here with anti-OLF1 and anti-OLF2. Antibody-mediated degradation of mutant myocilin is an orthogonal approach because it involves targeting OLF itself (21, 79).

There has been a long standing interest in gene delivery in the eye because the eye is an immune-privileged organ (80) that is attractive for a variety of drug delivery modalities such as viral vectors and nanoparticles (81). Our approach would be to deliver genetic material that encodes antibodies rather than directly delivering a ligand to a glaucoma target. Delivery to TM cells and continuous dosing will be a challenge but advances in in situ antibody expression technologies (82), which show promise for treating infectious diseases (83–85) and other diseases like cancer (86), support the concept of antiaggregation therapeutic antibodies that act in the ER. In the long-term, combined with early genotyping strategies to identify disease-causing myocilin mutations, OLF-targeting antibodies could lead to vision-saving therapies for millions of at-risk patients. More broadly, our study supports the further development of autophagy manipulation as a therapeutic strategy to treat proteostasis decline in the anterior segment, which contributes to IOP elevation in glaucoma (87). Finally, it may be possible to extend the application of our intracellular antibody strategy to other disease-causing ER-resident targets with unknown function and a gain-of-function pathogenesis.

## Methods

### OLF purification

WT myocilin OLF was expressed and purified as previously described (30, 88). See also Supplementary Methods.

### Mouse immunization, antibody phage display, and phage panning

Mouse immunization and antibody discovery was conducted largely as described previously (89). All protocols were approved by the University of Texas at Austin IACUC (AUP-2018-00092), and mice were handled in accordance with IACUC guidelines. A phage antibody library was constructed as described previously (89). Clones with good expression and specific binding to one or both OLFs were validated by the same ELISA with full dilution curves and sequenced. Clones exhibiting specific OLF binding, unique CDR sequence identity, high affinity, and high expression were prioritized and further characterized into full-length IgGs. See also Supplementary Methods.

### Recombinant hIgG1 production

Lead phage antibodies were converted into chimeric human IgG1 antibodies as described previously (89). See also Supplementary Methods.

### Antibody digestion into antigen binding fragments

See Supplementary Methods.

### Evaluation of antibody binding by ELISA

High binding plates (Corning) were coated with 1 μg/mL antigen in phosphate buffered saline (PBS) and incubated overnight at 4 °C. Coat antigens included: anti-c-myc antibody 9E10, SEC-purified monomeric human or mouse OLF, purified monomeric, or aggregated MBP-OLF isolated from the SEC void-volume fractions that exhibits hallmarks of amyloid (31). The next day, plates were blocked with PBS supplemented with 0.1% Tween-20 (PBS-T) and 5% dry nonfat milk for 1 h at room temperature. To rank purified antibodies based on binding affinity, human IgG1s or Fabs were serially diluted in blocking buffer (1:5) from 5 μg/mL. After 1 h, plates were washed and secondary antibodies (Table S3) were applied for an additional 1-h incubation. PBS-T + 1% dry nonfat milk was used as assay diluent and PBS-T was used for triplicate washing of plates in between incubation steps. All steps proceeded at room temperature unless indicated. Signals were developed using 50 µL/well 3,3’,5,5’-tetramentylbenzidine (TMB) solution (Thermo Scientific Pierce), quenched with an equal volume of 1 N HCl and the absorbance was measured at 450 nm using a Molecular Devices SpectraMax m5 or a Biotek Synergy 2 plate reader. Absorbance means and standard deviations were calculated and compared with controls using GraphPad Prism.

To assess antibody polyspecificity, plates were coated with a panel of chemically distinct antigens: lipopolysaccharides from *Escherichia coli* O111:B4 at 10 μg/mL, single-stranded DNA from calf thymus at 1 μg/mL, DNA from calf thymus at 1 μg/mL, insulin at 5 μg/mL, and cardiolipin at 50 μg/mL (all from Sigma). PBS was supplemented with 0.5% bovine serum albumin (BSA) and 0.1% Tween-20 and used as a blocking buffer for all plates except for cardiolipin which used PBS with 10% fetal bovine serum (FBS) for blocking. Antibodies of interest, including a control antibody 3H9 (GenBank accession: M18237 and M18234), which binds double stranded DNA and is known to be polyspecific (90), were serially diluted from Ref. (2) (Table S1).

### Bio-layer interferometry

Antibody equilibrium binding affinities (*K_D_* values) were measured using a ForteBio OctetRed instrument. First, OLF was biotinylated using EZ-Link Sulfo-NHS-Biotin (Thermo Scientific) using a 1:20 molar ratio according to the manufacturer's protocol. Biotinylation was confirmed by Western blot using streptavidin-horseradish peroxidase (HRP) (Fig. S3E, Table S1) and imaged using an iBright imaging system (Thermo Fisher Scientific). Streptavidin biosensors (Sartorius) were loaded with biotinylated-OLF at 20  μg/mL in (10 mM 4-(2-hydroxyethyl)piperazine-1-ethanesulfonic acid (HEPES), 150 nM NaCl, 3 mM EDTA, 1 mg/mL BSA, and 0.5% Tween-20, HBS-EBT) for 10 min. Biosensors were dipped into wells containing anti-OLF1 or anti-OLF2 Fab serially diluted 1:3 from 111.1 nM, with one biosensor dipped into HBS-EBT as a control. After association for 300 s, all biosensors were dipped into HBS-EBT for 300 s to monitor dissociation. The reference no antibody curve was subtracted from the other curves, and the ForteBio analysis software was used to globally fit a 1:1 Langmuir binding model, obtain kinetic rate constants for each binding interaction, and calculate standard deviations for each. Curves are representative of two biological replicates.

### Dot and Western blots

Dot and Western blots were performed as previously described (89) in at least two biological replicates with at least two analytical replicates. Membranes were probed with purified IgGs at either 0.5 µg/mL (anti-OLF1 and anti-OLF2) or 0.05 µg/mL (anti-OLF2). See also Table S1.

### Immunoprecipitation from HTM cell culture conditioned media

Conditioned media from primary human TM cell culture used in this experiment was a kind gift from Snider and Ethier (91). Immunoprecipitation was performed as previously reported (91). Antibodies were incubated with 15 mL of either conditioned human TM medium without enrichment or HBS (50 mM HEPES pH 7.5, 200 mM NaCl, 10% glycerol) as a control. Results represent at least two biological replicates for each IgG tested. See sequences in Table S1.

### Thioflavin-T aggregation assay

Aggregation of WT OLF in the presence of purified IgGs and Fabs was monitored by ThT fluorescence, similar to previous studies (32). See also Supplementary Methods.

### Hydrogen deuterium exchange mass spectrometry

SEC was performed to isolate WT OLF and Fab complexes (Fig. S13). Prior to SEC, WT OLF was incubated with Fab at 1:1 molar ratio on ice for 2 h. Fractionation was performed using a Superdex 75 GL column on an AKTA PURE (GE Healthcare) equilibrated with HEPES buffer (10 mM HEPES pH 7.2, 0.2 M NaCl) at 4 °C. Assessment of fractions was done with 12% SDS-PAGE analysis. Fractions containing WT OLF and Fab complex were pooled and concentrated to 30 μM using an Amicon filtration device (Millipore Sigma) with a 30-kDa molecular weight cutoff prior to HDX-MS experiments. Anti-OLF1 Fab and OLF did not retain complexation under these specific SEC conditions (Fig. S13).

HDX-MS experiments were performed on purified WT OLF in the presence and absence of antibodies. For anti-OLF2, WT OLF and Fab complex isolated with SEC was used for HDX-MS. For anti-OLF1, SEC fractions of OLF and fractions of Fab were isolated and pooled separately (Fig. S13). OLF was then incubated with anti-OLF1 Fab at 1:1 molar ratio overnight at 4 °C. All samples used for HDX-MS were in HEPES buffer (10 mM HEPES pH 7.2, 0.2 M NaCl). Samples were analyzed in triplicates using a Waters HDX system with nanoAcquity UPLC and Micromass Q-ToF Premier mass spectrometer (Waters Corp, Milford, MA, USA). Samples were mixed at 1:7 (v:v) ratio with D_2_O-containing buffer (10 mM phosphate, 99.9% D_2_O, pD 7.0) by an automated LEAP Technology pipetting robot for 10 to 10,000 s at 20 °C. Each time point was repeated five times. The exchange reaction was quenched with precooled quenching buffer (100 mM phosphate, 0.5 M tris(2-carboxyethyl)phosphine, 0.8% formic acid, 2% acetonitrile, pH 2.5) for 180 s at 1 °C. Quenched reactions were digested on a Waters Enzymate BEH Pepsin Column (2.1 × 30 mm) at 20 μL/min flow rate. Peptic fragments were separated using a 12-min acetonitrile gradient from 40 to 90% at 40 μL/min on a Waters ACQUITY UPLC BEH C18 column (1.7 µm, 1.0 × 100 mm) at 1 °C. For mass spectrometer analysis, positive ion mode was used for the electrospray ionization source and a reference lock-mass of Glu-Fibrinopeptide (Sigma-Aldrich, St Louis, MO, USA) was measured along with each sample as an internal control. The sequence of WT myocilin OLF was used for peptide identification in ProteinLynx Global SERVER (version 3.02). HDX-MS data were processed using DynamX (version 3.0) and mass assignment for each peptide at each time point was done manually. Mass assignments of a deviation greater than 0.2 Da were removed. The difference between the relative fractional uptakes for each residue was used to calculate HDX protection in the presence of the antibodies. This difference was mapped onto the X-ray crystal structure of WT myocilin OLF (PDB code: 4WXQ) using positive b-values (0–30% difference range). HDX-MS data presented in the text and figures were generated using DynamX.

### Immunofluorescence imaging of HTM cells

Immortalized HTM-1 cells (39) were routinely cultured in Gibco 1 g/L glucose DMEM supplemented with 10% FBS (Cytiva) and 1–2% penstrip (MP Biomedicals) according to consensus recommendations (38). For immunocytochemistry, cells (*n* = 2) were seeded at ∼90% confluence on glass coverslips (12 mm diameter, Fisher) coated with 2% gelatin (catalog number G1393-20ML, Sigma-Aldrich, St. Louis, MO, USA). The next day, media was changed to serum-free media prior to transfection. Transfection was performed with 2 μg total plasmid at 1:1 ratio of myocilin to antibody and 4:1 ratio of light chain to heavy chain. Plasmids were prepared in Lipofectamine 2000 (Invitrogen) at a ratio of 1 µg plasmid to 2.5 µL Lipofectamine 2000 mixed in serum-free media according to manufacturer's protocol. After 48 h, cells were fixed with 10% formalin (Fisher Healthcare) for 30 min at room temperature. After a subsequent 1× PBS (Gibco) wash, cell membranes were permeabilized with 0.03% Triton X-100 (VWR Health Sciences) for 30 min at room temperature. Slides were blocked in 5% milk for >1 h at 4 °C before incubating at 4 °C with primary antibodies >12 h (1:500 rat anti-FLAG (L5), Alexa Fluor 488, Invitrogen; 1:500 rabbit antimyocilin (Ab41552), Abcam; 1:500 mouse anti-FLAG (M2), Sigma). Following PBS wash, cells were incubated at 4 °C with 1:1,000 secondary antibodies (goat antirabbit IgG, Alexa Fluor 488, Invitrogen; goat antimouse IgG, Cyanine5, Invitrogen) for 1 h at room temperature. This step was skipped for cells incubated with the anti-FLAG (L5), Alexa Fluor 488 primary antibody. Following another PBS wash, cells were incubated with 1:1,000 Hoechst 33342 in in 1X assay buffer (catalog number ENZ51035K100, Enzo Life Sciences, Farmingdale, NY, USA) for 30 min at room temperature. Cells were washed before fixing coverslips with ProLongTM Diamond Antifade Mountant (Invitrogen) and allowed to cure for 24 h.

Three representative images/slide were taken at 40× magnification with a Leica DMB6 fluorescent microscope (Leica Microsystems, Wetzlar, Germany) and processed with Leica Application Suite X software. Zoomed insets (Fig. 3B) were created by objectively choosing 400 × 400 pixel fields of view from 40× magnification images in Fig. 3A. The brightness of the whole panel in Fig. 3A and B was adjusted from Fig. S9 following the removal of highly fluorescent GFP. Experiments were conducted with two independent replicates with one replicate using anti-FLAG (L5), Alexa Fluor 488 and another independent replicate with two technical replicate coverslips using antimyocilin rabbit (Ab41552) and anti-FLAG (M2) mouse.

### Myocilin secretion assay: cell culture

HEK293T cells (American Type Culture Collection) were grown and maintained in Dulbecco's modified Eagle medium (DMEM, Corning) supplemented with 10% FBS (Hyclone), and 1% penicillin–treptomycin–glutamine (Gibco) at 37 °C with 5% CO_2_. The plasmid for full-length WT myocilin used in cellular experiments was custom cloned in pcDNA 3.1 vector with a C-terminal FLAG tag (GenScript) as previously reported (92). The plasmid for full-length myocilin with the P370L mutation was produced by site-directed mutagenesis (QuikChange Lightning Mutagenesis Kit, Agilent). All sequences were verified by DNA sequencing (GenScript). The antibody heavy and light chain plasmids used for recombinant production of OLF1 and OLF2 were used. Prior to transfection, cells were plated at 70–80% confluency in cell culture treated 6-well plates (Thermo Scientific) and allowed to grow for 24 h. Transfection was performed with 2 μg total plasmid at 1:1 ratio of myocilin to antibody and 4:1 ratio of light chain to heavy chain. Plasmids were prepared in Lipofectamine 2000 (Invitrogen) at a ratio of 1 µg plasmid to 2.5 µL Lipofectamine 2000 mixed in serum-free Opti-MEM (Invitrogen) according to manufacturer's protocol. After 24 h following transfection, the medium was changed to either serum-free DMEM with 1% penicillin–streptomycin–glutamine for Western blot analysis, or fresh DMEM with 10% FBS and 1% penicillin–streptomycin–glutamine for dot blot analysis. Cells were harvested 48 h after transfection.

Tetracycline-responsive inducible human embryonic kidney (iHEK) cells (41) were grown and maintained in DMEM supplemented with 10% tetracycline approved FBS (Thermo Fisher Scientific, A4736401) and 1% penicillin–streptomycin–glutamine (Gibco) at 37 °C under 5% CO_2_. Cell selection was performed with supplementation of hygromycin B (200 µg/mL, Invitrogen) and G418 (100 µg/mL, Gibco). Cells were plated at 70–80% confluency in cell culture treated 6-well plates (Thermo Scientific) 24 h prior to transfection. Transfections of antibody light chain and heavy chain plasmids were performed as described above for HEK293T cells. Myocilin expression was induced with 1 µg/mL of doxycycline (Avantor) 24 h post-transfection. Doxycycline was prepared in either serum-free DMEM with 1% penicillin–streptomycin–glutamine for Western blot analysis, or fresh DMEM with 10% tetracycline approved FBS and 1% penicillin–streptomycin–glutamine for dot blot analysis. Control samples lacking induction were changed to fresh medium without doxycycline. Cells were harvested 24 h after induction/medium change (48 h post-transfection) or were further treated with inhibitors.

### Myocilin secretion assay: inhibitor treatment

Doxycycline-induced iHEK I477N cells were treated with MG-132 (Sigma-Aldrich) and Baf A1 (Sigma-Aldrich) 24 h after induction. Stocks of inhibitors in dimethylsulfoxide (DMSO) were diluted in cell media to 5 μM of MG-132 and 100 nM of Baf A1 for cell treatment. The same volume of DMSO without inhibitors was added for the vehicle control samples. Cells were harvested 24 h post-treatment (or 72 h after transfection).

### Myocilin secretion assay: cellular fractionation

Cell media were collected and treated with a protease inhibitor cocktail containing one tablet of cOmplete EDTA-free protease inhibitor (Roche) and 1% (v/v) of phosphatase inhibitor cocktails II and III (Sigma-Aldrich) at a 1:100 ratio of media to protease inhibitor cocktail. Cell media of disease-causing myocilin mutants (with low expression of secreted myocilin) were concentrated 10-fold with an Amicon filtration device (Millipore Sigma) with a 10-kDa molecular weight cutoff for immunoblotting. Cell media of WT myocilin (with high expression of secreted myocilin) were used directly for immunoblotting without concentrating. Adherent cells were scraped and lysed with Triton-X 100 lysis buffer (100 mM Tris-HCl pH 7.4, 3 mM ethylene glycol bis(β-aminoethyl ether)-N,N,N′,N′-tetraacetic acid (EGTA), 5 mM MgCl_2_, 1 mM phenylmethylsulfonyl fluoride, 0.5% Triton-X 100) supplemented with 1% (v/v) of the protease inhibitor cocktail. Cells were lysed overnight at −20 °C. The following day, cell lysates were thawed on ice and centrifuged at 13,000 × g for 10 min. The cellular soluble fraction (supernatant) was separated from the insoluble fraction. The insoluble fraction was resuspended and re-pelleted 3 times with 200 μL of ice-cold PBS (Gibco), then were resuspended in 300 μL of 2× Laemmli buffer with 5% (v/v) β-mercaptoethanol and sonicated with a rod sonicator (Qsonica Q125) for 8–10 min with 10-s on/off pulses at 50% amplitude.

Total protein of the cell media and soluble fraction was quantified using BCA assay (Pierce) according to the manufacture's protocol. For Western blot analysis, cell media and soluble (supernatant) samples were prepared in equal protein concentration (0.5–6 μg/μL) within each fraction and within each experiment in a final 1× Laemmli buffer containing 5–10% (v/v) β-mercaptoethanol. Samples were heated at 95 °C for 15–30 min and loaded on 4–15% mini-PROTEAN TGX precast protein gels (Bio-Rad) in equal protein quantity within each fraction and within each experiment. Insoluble samples were spiked with additional 3 μL of β-mercaptoethanol and loaded on gels in equal volume (14–20 μL) per lane. For dot blot analysis, cell media were prepared in equal protein concentration within each experiment in nuclease-free water.

For Western blot analysis, gels were transferred to methanol-activated polyvinylidene fluoride (PVDF) membranes using the Trans-blot Turbo transfer system (Bio-Rad). For dot blot analysis, cell media samples were deposited in equal protein quantities onto Amersham Protran nitrocellulose membranes (Sigma-Aldrich) soaked in TBS buffer (20 mM Tris-HCl, 500 mM NaCl pH 7.5) using a Bio-Dot microfiltration apparatus (Bio-Rad). Downstream Western and dot blot steps were performed as described above. Membranes were incubated with 1:1,000 dilution of primary antibodies (Table S3) overnight at 4 °C, followed by incubation with 1:2,500 dilution of secondary antibodies (Table S1). Blots were visualized using a ChemiDoc MP Imaging System (Bio-Rad). Results for cellular secretion assays represent at least two biological replicates.

Quantification of protein bands on Western blots was performed using ImageJ (93–95). Measurements of band density were normalized to the vehicle control sample (no-IgG control, DMSO-treated control) within each experimental treatment. The mean of the normalized density of biological replicates was calculated and reported as relative density using GraphPad Prism. Statistical significance was determined with two-tailed unpaired t-test.

### Flow cytometry

For analysis of protein aggregation, cell samples of HEK293T transfected with P370L and either anti-OLF1 or anti-OLF2 antibodies were stained with PROTEOSTAT Aggresome Detection kit (Enzo Life Sciences #51035-K100). Cell culture, transfection, and inhibitor treatment were performed as described above. Cells were collected 24 h after inhibitor treatment (48 h after transfection). For cell collection, adherent cells were dissociated using cell medium containing 0.05% Trypsin with 0.5 mM EDTA. Cells were washed with 1× PBS and fixed in 10% formalin for 30 min at room temperature. Cells were then stained with the PROTEOSTAT Aggresome Detection Reagent 1:10,000 dilution according to manufacturer's protocol for flow cytometry. Stained cells were analyzed on a BD FACS Melody flow cytometer. PROTEOSTAT signal was measured with a 488 nm laser with the PE-Cy7 channel with a 783/56 filter and 752 long pass filter. Data were collected using FACS Chorus software and analyzed with FlowJo (v10.8.1). PROTEOSTAT positive cells were selected and compared by mean fluorescence intensity (MFI) (Fig. S12). APF was then calculated as APF = 100 × (MFI treated − MFI control)/MFI treated.

### Immunofluorescence imaging of HEK293T cells

Glass microscope cover slips (12 mm diameter, Fisher) placed in 24-well plates were soaked in poly-L-lysine (ScienCell) for 30 min and excess solution was aspirated off. HEK293T cells were seeded at 70–90% confluency into 24-well plates containing the coated microscope cover slips and then incubated overnight. The following day, cells were transfected as described above. After 48 h post-transfection, cells were fixed and blocked with 2% BSA (Sigma) diluted in 1× PBS. Primary antibodies (Table S3) diluted in 0.1% BSA/PBS were added and incubated for 3 h at room temperature. After primary antibody incubation, coverslips were washed three times with PBS. Secondary antibodies (Table S3) diluted in 0.1% BSA/PBS solution were added and incubated for 45 min at room temperature. After secondary antibody incubation, coverslips were again washed three times with PBS. Lastly, coverslips were stained with Hoeschst 33342 and PROTEOSTAT Aggresome Detection Reagent (Enzo Life Sciences #51035-K100) which was diluted 1:1,000 and 1:2,000 respectively in 1× assay buffer and incubated for 30 min at room temperature. Coverslips were then washed three times with PBS and stained with the PROTEOSTAT Aggresome Reagent diluted 1:2,000 as described above using the manufacturers protocol for adherent cells. Coverslips were then mounted using Antifade Gold Mounting Reagent (Invitrogen) and allowed to cure for 24 h. Fluorescence images were captured using tile scans on a laser scanning confocal microscope (ZEISS LSM 700 with AxioObserver with 63 × 1.4 oil immersion objective). Experiments were conducted with two biological replicates with two technical replicate coverslips (*n* = 2,2); images presented in figures are representative of both replicates.

Fields of view from tile scans were chosen objectively with 512 × 512 pixel sizes. Images were then processed using Zen Lite software (Carl Zeiss). Intensity for each channel (calnexin, myocilin, or PROTEOSTAT) was scaled to the brightest field of view observed. Gamma correction was then performed to optimize background staining for myocilin and PROTEOSTAT channels.

Intensity analysis of each channel was calculated with ImageJ (93–95) using measured mean gray value using *n* = 3 independent fields of view on each coverslip and protein signals were normalized by the calnexin signal. Statistical significance was measured with one-way ANOVA using Dunnett's multiple comparisons test for post-analysis. Outliers were removed using the ROUT test. Zoomed images were created by cropping 400 × 400px regions representative of the field of view.

## Supplementary Material

pgae556_Supplementary_Data

## Data Availability

The authors declare that all data supporting the findings of this study are available within the article and its supplementary information files. Sequences of the novel antibodies reported (anti-OLF1, anti-OLF2) are provided in Table S1. The HDX-MS was deposited to the ProteomeXchange Consortium repository.
