# Supplementary material for: Antibody-mediated clearance of an ER-resident aggregate that causes glaucoma
Source: PNAS Nexus. 2024 Dec 10;4(1):pgae556. doi: 10.1093/pnasnexus/pgae556 (PMC11670252; doi:10.1093/pnasnexus/pgae556)
Supplement: pgae556_Supplementary_Data [file pgae556_supplementary_data.pdf]

Supplementary Information

Antibody-mediated clearance of an ER-resident aggregate that causes glaucoma

Minh Thu Ma[1], Ahlam N. Qerqez[2], Kamisha R. Hill[1], Laura R. Azouz[2], Hannah A. Youngblood[1], Shannon E. Hill[1], Yemo Ku[1], Donna M. Peters[3], Jennifer A. Maynard[2], Raquel L. Lieberman[1]

[1] School of Chemistry & Biochemistry, Georgia Institute of Technology, 901 Atlantic Drive NW Atlanta, GA 30332 USA

[2] Department of Chemical Engineering, and [3]Department of Molecular Biosciences University of Texas at Austin, Austin, TX 78712 USA

[3] Department of Pathology & Laboratory Medicine and Department of Ophthalmology & Visual Sciences, University of Wisconsin School of Medicine and Public Health, Madison, WI 53705, USA.

Supplemental Methods
Table S1. Anti-OLF1 and antiOLF2 sequences
Table S2. Binding kinetics of anti-OLF1 and anti-OLF2 to human OLF as measured by bio-layer interferometry.
Table S3. Commercial antibodies used in this study.
Figure S1. Identification of OLF antibodies.
Figure S2. Uncropped blots and SDS-PAGE gels for anti-OLF1 and anti-OLF2 corresponding to data shown in Figures 2B,D.
Figure S3. Characterization of initial antibody candidates and analysis of anti-OLF1 and anti-OLF2 antigen specificity.
Figure S4. ThT fluorescence aggregation assays for WT OLF in the presence of candidate antibodies.
Figure S5. HDX-MS data for unbound and antibody-bound WT OLF.
Figure S6. Representative mass spectra of anti-OLF1 Fab binding epitope showed in Figure 2E after 10,000-second deuterium exchange.
Figure S7. Representative mass spectra of anti-OLF2 Fab binding epitope showed in Figure 2F after 10,000-second deuterium exchange.
Figure S8. Representative deuterium uptake graphs (middle) and mass spectra (left and right) of anti-OLF2 Fab binding epitope indicated in Figure 2F after 10,000-second deuterium exchange.
Figure S9. Immunofluorescence imaging of HTM-1 cells.
Figure S10. Full immunoblots of secretion assays for HEK293T cell models and quantitation data.
Figure S11. Tracking of anti-OLF1, anti-OLF2, and 2E9 across cellular experiments.
Figure S12. Raw data used in flow cytometry analysis.
Figure S13. Size-exclusion chromatography and fractional SDS-PAGE analysis of WT OLF complexed with A, anti-OLF1 Fab and B, anti-OLF2 Fab.
Supporting References.

Supplemental Methods

Recombinant expression and purification of OLF. Wild-type (WT) myocilin OLF was expressed and purified as a maltose binding protein (MBP) fusion with a tobacco etch virus (TEV) cleavage site as previously described (1, 2). Hexa-His-tagged TEV protease was produced in-house (3). Protein purity was determined via 12% SDS-PAGE analysis using either Coomassie staining or stain-free Tris-glycine gels. Gels were imaged with a ChemiDoc MP Imaging System (Bio-Rad).

Mouse immunization, antibody phage display, and phage panning. Mouse immunization and antibody discovery was conducted largely as described previously (4). BALB/c mice (6 weeks old, n = 3) were immunized subcutaneously with 7.5 μg purified human OLF protein in Freund's complete adjuvant. They were boosted three weeks later with 7.5 μg mouse OLF protein and then again two weeks later with 7.5 μg each human and mouse OLF protein, both times with Freund's incomplete adjuvant. Two weeks after the final boost, mice were sacrificed, their spleens harvested and stored in 1 mL RNAlater solution at -80 °C. Sera were used in ELISA to determine the serum antibody titers against human and mouse OLF. All protocols were approved by the University of Texas at Austin IACUC (AUP-2018-00092), and mice were handled in accordance with IACUC guidelines.

A phage antibody library was constructed as described previously (4). The library was panned first against anti-c-myc monoclonal antibody (9E10; BioXCell, **Table S1**), and then against human OLF. This round 2 output was expanded and half the phage panned against human OLF again to generate output HH, while the other half was panned against mouse OLF to identify antibodies binding conserved epitopes to generate output HM. The round 3 outputs were

expanded and again half the phage were panned against human OLF and half against mouse OLF to generate four final pools of clones named HHH, HMH, HHM and MMM based on the order of panning against human (H) or mouse (M) OLF (**Supp. Fig. 1B**). Clones from each pool were used to infect XL1-Blue *E. coli* for monoclonal phage production in 96 wells plates followed by ELISA screening against anti-c-myc antibody 9E10 (**Table S1**), human OLF, mouse OLF or blocked wells with anti-M13-HRP (Santa Cruz, **Table S1**) detection. Clones with good expression and specific binding to one or both OLFs were validated by the same ELISA with full dilution cures and sequenced. Clones exhibiting specific OLD binding, unique CDR sequence identity, high affinity, and high expression were prioritized and further characterized into full length IgGs.

Recombinant hIgG1 production. Lead phage antibodies were converted into chimeric human IgG1 antibodies by cloning the V_H domains with primers into the IgG-AbVec vector using *AgeI* and *SalI* (NEB) digestion, and the V_L domains with primers into the Igk-AbVec vector using *AgeI* and *BsiWI* (NEB) digestion, as described previously (4). Plasmids were transfected into ExpiCHO cells using ExpiFectamine (Thermo Fisher Scientific) according to the manufacturer's standard protocol, with a 4:1 ratio of light to heavy chain vector. Cells were maintained in ExpiCHO Expression Media (Thermo Fisher Scientific) at 37 °C with shaking for transient expression, with supplementation with Expifectamine CHO Enhancer and Expifectamine CHO Feed after 1 day. After 1 week, cell culture supernatant was collected and applied to a HiTrap protein A column which had been equilibrated with a 25 mM Tris, 25 mM sodium chloride pH 7.4 binding buffer. After washing, recombinant antibody was eluted by applying a 100 mM sodium citrate and 50 mM sodium chloride pH 3.0 elution buffer. Protein

concentrations were measured using Nanodrop 200, and purity was assessed by SDS-PAGE on a 4-20% gel (Bio-Rad) with Coomassie staining.

Antibody digestion into antigen binding fragments. Antibodies (10 mgs) were buffer exchanged into digestion buffer (20mM sodium phosphate, 10mM EDTA at pH 7.0, with cysteine-HCl added to a final concentration of 20mM just prior to use). A slurry of immobilized papain resin (Thermo Scientific) was washed with digestion buffer twice and combined with antibody incubated at 37 °C with end-over-end mixing. After 5 hours, the aqueous phase was collected and resin washed twice with phosphate-buffered saline (PBS; 10 mM Na_2H/KH_2PO_4, 200 mM NaCl, pH 7.2) for combination with the flow-through. This was incubated with protein A resin and mixed end-over-end at room temperature for 30 minutes to capture intact IgG and Fc fragments. The Fab domains were recovered from the aqueous phase, the protein A resin washed once with PBS, and combined with the flow-through for concentrating using an Amicon filtration device (Fisher Scientific) with a 10-kDa cut-off to ~1 mg/ml. Protein concentrations and purity were assessed as above.

Thioflavin-T aggregation assay. Aggregation of WT OLF in the presence of purified IgGs and Fabs was monitored by ThT fluorescence, modified from previous studies (5). Prior to ThT aggregation assay, WT OLF was incubated with the antibodies for 1 hour on ice in PBS with the following concentrations: 30 μM OLF and 30 μM IgG (1:1), 30 μM OLF and 15 μM IgG (2:1), 15 μM OLF and 7.5 μM Fab (2:1), to account for bivalency in antibody binding to OLF. A 1 mg/mL stock of ThT in deionized water was diluted with PBS to a 20 μM working solution. This working solution was added to assays for a final concentration of 10 μM ThT per well. Samples were prepared as master mixes (75 μL – 150 μL) in 1.5 mL Eppendorf tubes, transferred to 96-well black FLOUTRAC microplates (Greiner), and sealed with clear

MicroAmp PCR film (Applied Biosystems). Fluorescence was measured every 10 minutes for 96 hours at 42 °C using an BioTeK Synergy 2 plate reader at excitation wavelength of 440 nm (slit width 30 nm) and emission wavelength of 485 nm (slit width 20 nm). The average intensity of at least 2 analytical replicates per sample was calculated. The average initial intensity (at t = 0 minute) was subtracted from the average intensity for each time point of each sample. Resulting intensities at 480 nm were plotted using GraphPad Prism.

At the conclusion of the assays, samples were transferred to 1.5 mL microcentrifuge tubes, and centrifuged at 13,000 x g for 10 minutes. The supernatant was decanted carefully and pellets were washed 3 times via resuspension with 200 µL PBS and centrifugation at 13,000 x g for 10 minutes. The samples of the pellets, initial supernatants, and supernatants from the last wash step were subjected to 12% SDS-PAGE analysis using either 2× Laemmli buffer or 2× Laemmli buffer supplemented with 5% (v/v) β-mercaptoethanol, with either Coomassie staining or using stain-free gels and visualized with a ChemiDoc MP Imaging System (Bio-Rad).

Table S1. Anti-OLF1 and antiOLF2 sequences

Antibody	Heavy	Light
**Anti-OLF1**	**DNA sequence:**<br>CAGGTCCAGCTCCAGCAATCTGGACCTGAGCTGGTGAAGCCTGGG<br>GCTTCAGTGAAGATTTCCTGCAAGACTTCTGGATACACTTTCACT<br>GAAAACACCATGCACTGGGTGAGGCAGAGCCATGGAAAGAGCCTT<br>GAGTGGATTGGAGGTATTCATCCTAACAATATTGGTAGTACCTAC<br>AACCAGAAGTTCAAGGGCAAGGCCACATTGACTGTAGACAAGTCC<br>TCCAGCACAGCCTACATGGAACTCCGCAGCCTGACATCTGAAGAT<br>TCTGCAGTCTATTACTGTACAAGAGGGGCTACGGCCCCGTTTGCT<br>TACTGGGGCCAAGGGACTCTGGTCACTGTCTCTGCG<br>**Protein sequence:**<br>QVQLQQSGPELVKPGASVKISCKTSGYTFTENTMHWVRQSHGKSL<br>EWIGGIHPNNIGSTYNQKFKGKATLTVDKSSSTAYMELRSLTSED<br>SAVYYCTRGATAPFAYWGQGTLVTVSA	**DNA sequence:**<br>GATATTGTGATGACCCAAACTCCACTCTCCCTGCCTGTCAGTCTTGGAGA<br>TCAAGCCTCCATCTCTTGCAGATCTAGTCAGAGCATTGTACATAGTAATG<br>GAAACACCTATTTACATTGGTACCAGCAGAAGTCAGGCCAGTCTCCAAAG<br>CTCCTGATCTACAAAGTTTCCAACCGATTTTCTGGGGTCCCAGACAGGTT<br>CAGTGGCAGTGGATCAGGGACAGATTTCACACTCAAGATCAGCAGAGTAG<br>AGGCTGAGGATCTGGGAGTTTATTTCTGCTCTCAAAGTACACATGTTCCT<br>CCGACGTTCGGTGGAGGCACCAAGCTGGAAATCAAACGT<br><br>**Protein sequence:**<br>DIVMTQTPLSLPVSLGDQASISCRSSQSIVHSNGNTYLHWYQQKSGQSPK<br>LLIYKVSNRFSGVPDRFSGSGSGTDFTLKISRVEAEDLGVYFCSQSTHVP<br>PTFGGGTKLEIKR
**Anti-OLF2**	**DNA sequence:**<br>GAAGTGAAGCTGGTGGAGTCAGGACCTGGCCTGGTGGCGCCCTCA<br>CAGAGCCTGTCCATCACATGCACCGTCTCAGGGTTCTCGTTAAGA<br>AATTATGGTGTACACTGGTTTCGCCAGCCTCCAGGAAAGGGTCTG<br>GAGTGGCTGGCAGTGACATGGAGTGATGGAAGCACAACCTATAAT<br>TCAGTTCTCAAATCCAGACTGAGCATCAGCAAGGACAACTCCAAG<br>AGCCAAGTTTTCTTAAAAATGAACAGTCTCCAAAGTGATGACACA<br>GCCATGTATTACTGTGCCAGAACCCTGAATCTTTATAGGTACGAC<br>GGTATGGACTACTGGGGTCGAGGAACCTCAGTCACCGTCTCCTCG<br>**Protein sequence:**<br>EVKLVESGPGLVAPSQSLSITCTVSGFSLRNYGVHWFRQPPGKGL<br>EWLAVTWSDGSTTYNSVLKSRLSISKDNSKSQVFLKMNSLQSDDT<br>AMYYCARTLNLYRYDGMDYWGRGTSVTVSS	**DNA sequence:**<br>GATATTGTGATGACTCAGTCTCCACTCTCCCTGCCTGTCAGTCTTGGAGA<br>TCAAGCCTCCATCTCTTGCAGATCTAGTCAGAGCCTTGTACACAGTAATG<br>GAAACACCTATTTACATTGGTACCTGCAGAGGCCAGGCCAGTCTCCAAAG<br>CTCCTGATCTACAAAGTTTCCAACCGATTTTCTGGGGTCCCAGACAGGTT<br>CAGTGGTAGTGGATCAGGGACAGATTTCACACTCAAGATCAGCGGAGTGG<br>AGGCTGAGGATCTGGGAGTTTATTTCTGCTCTCAAAGTACACATGTTCCT<br>CTCACGTTCGGTGCTGGGACCAAGCTGGAGCTGAAACGT<br><br>**Protein sequence:**<br>DIVMTQSPLSLPVSLGDQASISCRSSQSLVHSNGNTYLHWYLQRPGQSPK<br>LLIYKVSNRFSGVPDRFSGSGSGTDFTLKISGVEAEDLGVYFCSQSTHVP<br>LTFGAGTKLELKR

Table S2. Binding kinetics of anti-OLF1 and anti-OLF2 to human OLF as measured by bio-layer interferometry.

	k_{on} (10^5 $M^{-1}s^{-1}$)	k_{off} (10^{-3} s^{-1})	K_D (nM)	Full χ^2	Full R^2
**Anti-OLF1 Fab**	1.83 ± 0.02	3.35 ± 0.01	18.3 ± 0.18	0.6646	0.9939
**Anti-OLF2 Fab**	2.98 ± 0.03	1.67 ± 0.01	5.61 ± 0.07	0.2697	0.9899

Table S3. Commercial antibodies used in this study.

Antibody	Identification	Epitope & Clonality	Experiment & dilution
Mouse anti-c-myc 9E10	BioXCell Cat# BE0238 RRID: AB_2687720	Human c-myc (monoclonal)	Panning (2 μg/ml)
Mouse anti-M13 RL-ph1 HRP	Santa Cruz Cat# sc-53004 RRID: AB_673750	anti-g8p (monoclonal)	ELISA (1:2000)
Goat anti-human IgG Fc HRP	Southern Biotech Cat# 2048-05 RRID: AB_2795688	Human IgG Fc region (polyclonal)	ELISA (1:2000)
Goat anti-human kappa*HRP	Southern Biotech Cat# 2060-05 RRID: AB_619883	Human kappa light chain (polyclonal)	ELISA (1:2000) Immunoblotting of purified proteins (1:5000)
Streptavidin-HRP	BC Biosciences Cat# 554066	n/a	Western blot (1:2000)
Commercial rabbit anti-myocilin (ab41552)	Abcam Cat# ab41552 RRID: AB_776605	Myocilin far N-terminal within residues 25-46 conjugated to KLH (polyclonal)	Immunoprecipitation (1:1000) Fluorescent microscopy (1:500)
Goat anti-rabbit*HRP	Avantor Cat# 95058-730 RRID: AB_2934079	Rabbit IgG (polyclonal)	Immunoprecipitation (1:1000)
Commercial mouse anti-myocilin antibody	R&D Systems Cat# MAB3446 RRID: AB_2148649	Human myocilin LZ within residues 112–185 (monoclonal)	Immunoblotting of cell culture samples (1:1000)
Rabbit anti-β-actin	Cell Signaling Technology Cat# 4970 RRID: AB_2223172	Human β-actin (monoclonal)	Immunoblotting of cell culture samples (1:1000)
Goat anti-mouse Starbright Blue 520	Bio-Rad Cat# 12005866 RRID: AB_2934034	Mouse IgG (polyclonal)	Immunoblotting of cell culture samples (1:2500)
Goat anti-rabbit Starbright Blue 700	Bio-Rad Cat# 12004161 RRID: AB_2721073	Rabbit IgG (polyclonal)	Immunoblotting of cell culture samples (1:2500)
Rabbit anti-calnexin	Invitrogen Cat# PA5-34754 RRID: AB_2552106	Calnexin (polyclonal)	Confocal microscopy (1:200)
Mouse anti-FLAG	Cell Signaling Cat# 8146 RRID: AB_10950495	DYKDDDDK Tag (9A3) (monoclonal)	Confocal microscopy (1:200)
Goat anti-rabbit Alexa Fluor 488	Thermo Fisher Cat# A-11034 RRID: AB_2576217	Rabbit IgG (polyclonal)	Confocal microscopy (1:1000)
Goat anti-mouse Cyanine5	Thermo Fisher Cat# A48255 RRID: AB_2534033	Mouse IgG (polyclonal)	Confocal microscopy (1:1000)
Rat anti-FLAG Alexa Fluor 488	Invitrogen Cat# MA1-142-A488 RRID: AB_2610653	Rat IgG (monoclonal)	Fluorescent microscopy (1:500)
Mouse anti-FLAG	Sigma Cat# F3165-1MG RRID: AB_259529	Mouse IgG (monoclonal)	Fluorescent microscopy (1:500)

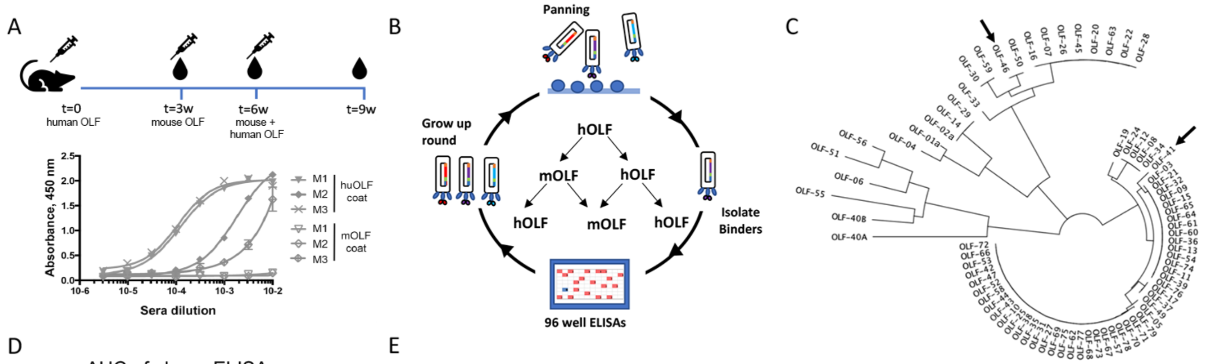

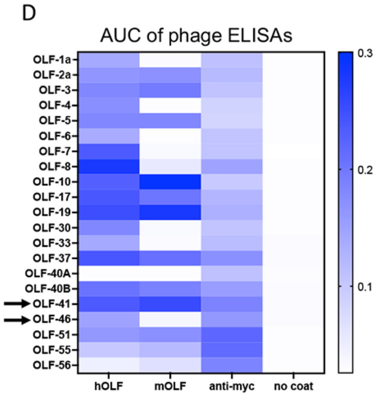

E

Clone	CDRL1	CDRL2	CDRL3		CDRH1	CDRH2	CDRH3	Clone Frequency %
OLF-2a	RSSQSIVHSNGNTYLE	KVSNRFS	FQGSHVPLT		GFTFSSYAMS	TISSGGSYTYYPDSVKG	LGGGIFAY	3.75
OLF-4	RSSQSLVHSNGNTYLH	KVSNRFS	SQTTHVPLT		GFTFRSTAMS	SISGRGNILYPDSVEG	YDSGAMDY	1.25
OLF-6	KASQNVGTNVA	SASYRYS	QQYNSYPLT		GYTFTSSYWMH	EINPSNGRTNYNEKFKS	GNYY	1.25
OLF-40B	KASQNVGTAVA	SASNRYT	QQYSSYPLT		GYSFTSY	MIDPSDSETRLNQKFKD	LGFKDY	1.25
OLF-41	RSSQSIVHSNGNTYLH	KVSNRFS	SQSTHVPPT		GYTFTENTMH	GIHPNNIGSTYNQKFKG	GATAPFVY	1.25
OLF-46	RSSQSLVHSNGNTYLH	KVSNRFS	SQSTHVPLT		GFSLRNYGVH	VTWSDGSTTYNSALKS	TLNLYRYDGMDY	1.25

Fig. S1. Identification of OLF antibodies. (**A**), Mouse serum titers against human and murine OLF after the second booster immunization, as assessed by ELISA. (**B**), Phage panning strategy to select for human (hOLF)/ mouse (mOLF) cross-reactive antibodies. (**C**), Phylogenetic tree showing the sequence relatedness among all unique, human-myocilin reactive sequence families identified. (**D**), Heat map summarizing antibody binding profiles for human and mouse OLF, with anti-c-myc capture used to assess scFv display level and uncoated wells used to assess non-specific binding. Each cell depicts the area-under-the-curve (AUC) for an eight-point dilution curve. (**E**), Clones representing sequence families with distinct OLF binding profiles were expressed as recombinant human IgG1 proteins for full biochemical characterization. Shown are their CDR amino acid sequences and the frequency at which these clones were observed in the initial pool of selected antibodies. Note, OLF-41 and OLF-46 were renamed OLF-1 and OLF-2, respectively, in future figures.

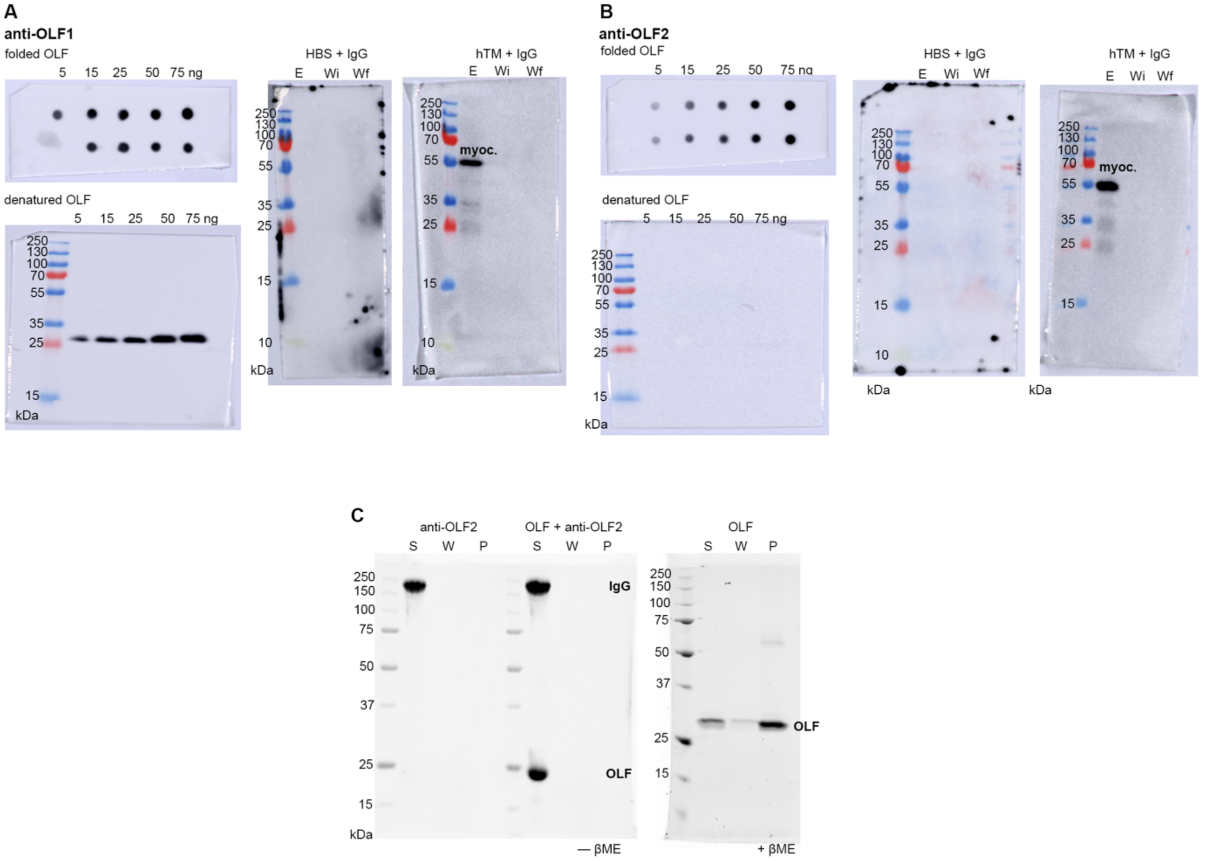

Fig. S2. Uncropped blots and SDS-PAGE gels for anti-OLF1 and anti-OLF2 corresponding to data shown in Figures 2B,D. (**A**), Anti-OLF1 and (**B**), anti-OLF2 were assessed: Left: against nanogram quantities of folded (dot blot, two analytical replicates per blot) and denatured (Western blot) of WT human OLF. Right: ability to immunoprecipitate endogenous myocilin. For immunoprecipitation, both antibodies were incubated in either buffer control (HBS, 50 mM HEPES pH 7.5, 200 mM NaCl, 10% glycerol) or spent hTM media. Protein A/G resin was washed 5 times with HBS before elution. Samples for initial wash step (Wi), final wash step (Wf), and elution (E) were analyzed with Western blot. (**C**), SDS-PAGE (±βME) analysis at the conclusion of ThT aggregation assay. Assay samples were harvested and the pellets were washed to generate the soluble (S), final wash (W), and insoluble (P) samples (see Methods). See Table S1 for list of commercial antibodies used for immunoblotting.

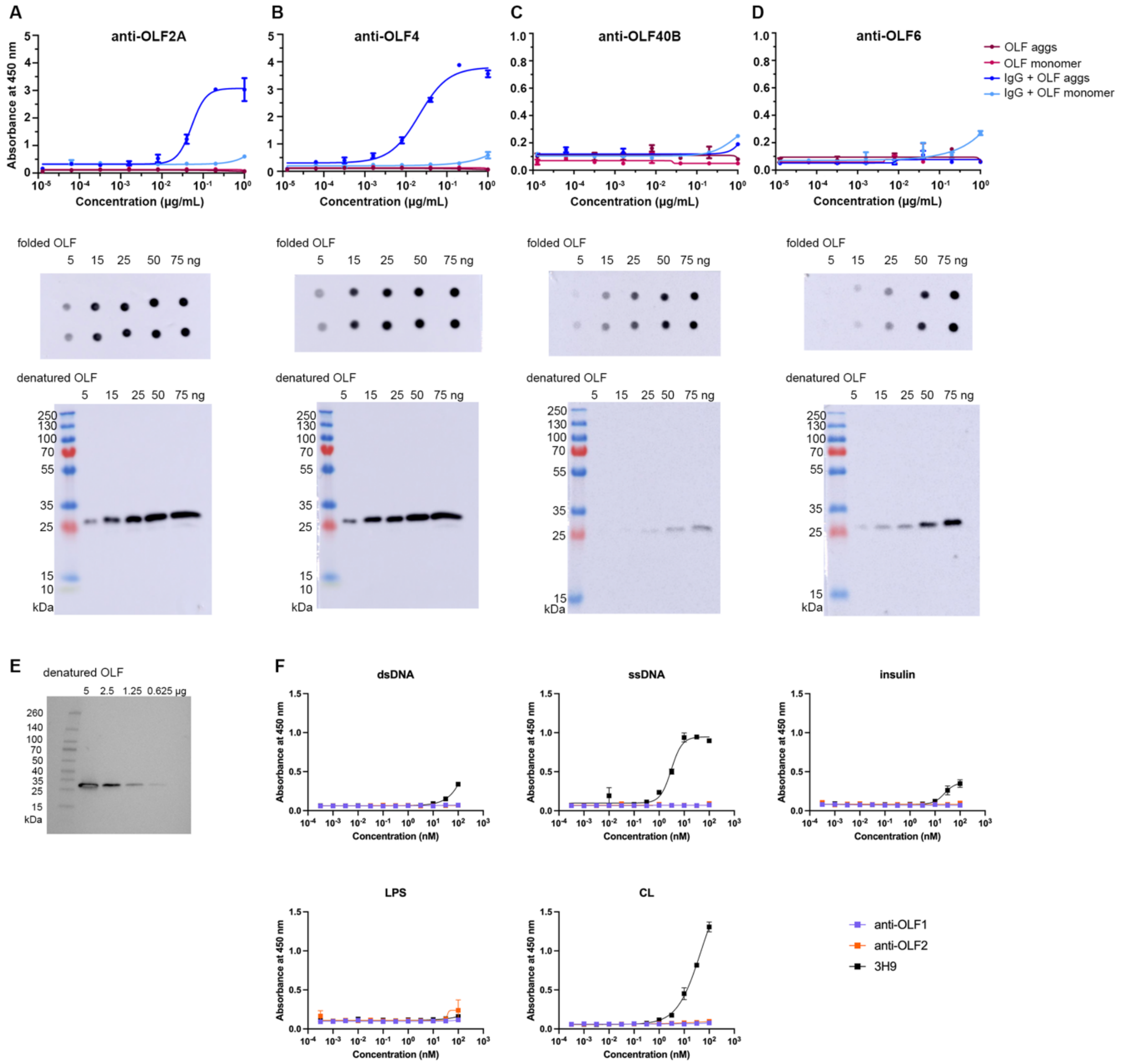

Fig. S3. Characterization of initial antibody candidates and analysis of anti-OLF1 and anti-OLF2 antigen specificity. (**A-D**), ELISA against monomeric OLF and SEC-isolated cellular OLF aggregates (top, see also Methods) indicate binding preference of anti-OLF2A and anti-OLF4 hIgG1s to soluble aggregates, and low binding affinity of anti-OLF40B and anti-OLF6 hIgG1s to both folded and aggregated OLF species. Dot blot and Western blot analysis (middle and bottom) against respective nanogram quantities of folded and denatured human OLF reveal no clear binding selectively for all 4 hIgG1s. (**E**), Western blot against biotinylated and denatured OLF using Streptavidin-HRP to show successful biotinylation. (**F**), ELISA against lipopolysaccharides from *Escherichia coli* O111:B4 (LPS), single-stranded deoxyribonucleic acid from calf thymus (ssDNA), deoxyribonucleic acid from calf thymus (dsDNA), insulin, and cardiolipin (CL). No binding was detected for anti-OLF1 and 2 even at 100 nM concentration of antigens, showing high specificity of both antibodies. Data is representative of two independent experiments. See Table S1 for list of secondary antibodies used.

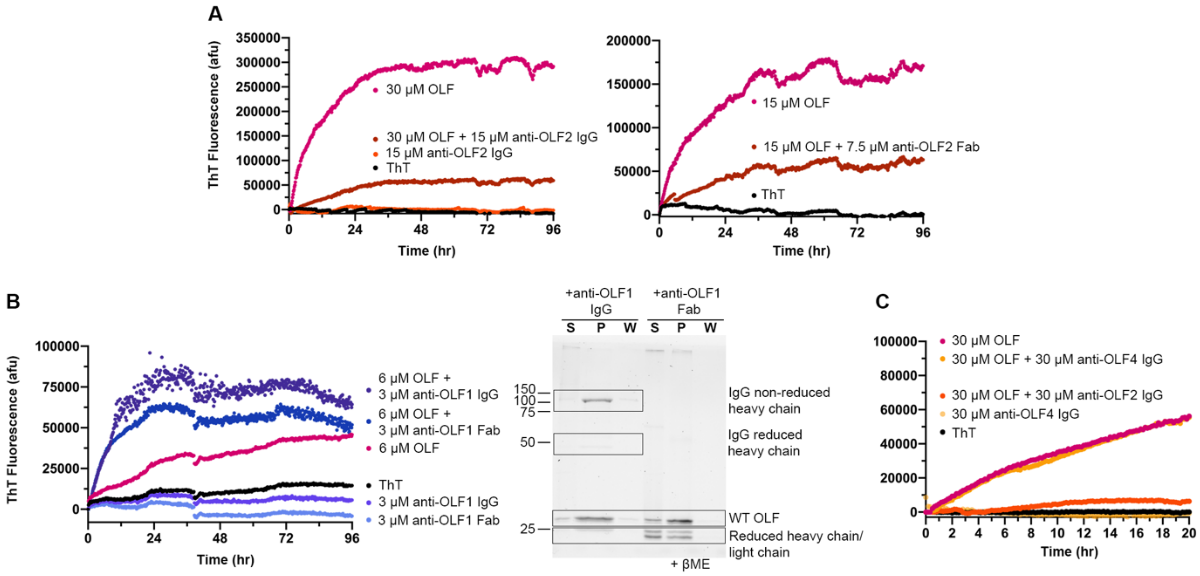

Fig. S4. ThT fluorescence aggregation assays for WT OLF in the presence of candidate antibodies. All ThT assays were performed at 42 °C. (**A**), ThT aggregation assays of OLF incubated with anti-OLF2 IgG or Fab to assess antibody bivalency in aggregation inhibition. At 2:1 OLF to IgG molar ratio (or 1:1 antigen to antibody binding site, left) and 2:1 OLF to Fab molar ratio (or 2:1 antigen to antibody binding site, right), anti-OLF2 is able to inhibit OLF aggregation but not to the full extent seen at 1:2 antigen to antibody binding site in Figure 2D, where fluorescence of OLF+IgG is similar to that of ThT control sample. (**B**), ThT aggregation assay (left) and subsequent SDS-PAGE analysis (right) of OLF incubated with anti-OLF1 IgG at 2:1 OLF to IgG molar ratio (or 1:1 antigen to antibody binding site), and with anti-OLF1 Fab at 2:1 OLF to Fab molar ratio (or 2:1 antigen to antibody binding site). SDS-PAGE lanes: soluble (S), insoluble (P), final wash (W) (see Methods). (**C**), ThT aggregation assay of OLF incubated with either anti-OLF2 IgG or anti-OLF4 IgG, both at 1:1 OLF to IgG molar ratio. While incubation with anti-OLF2 reduces levels of OLF aggregation to near ThT control sample, anti-OLF4 has no effect on OLF aggregation. Data are representative of at least two biological replicates.

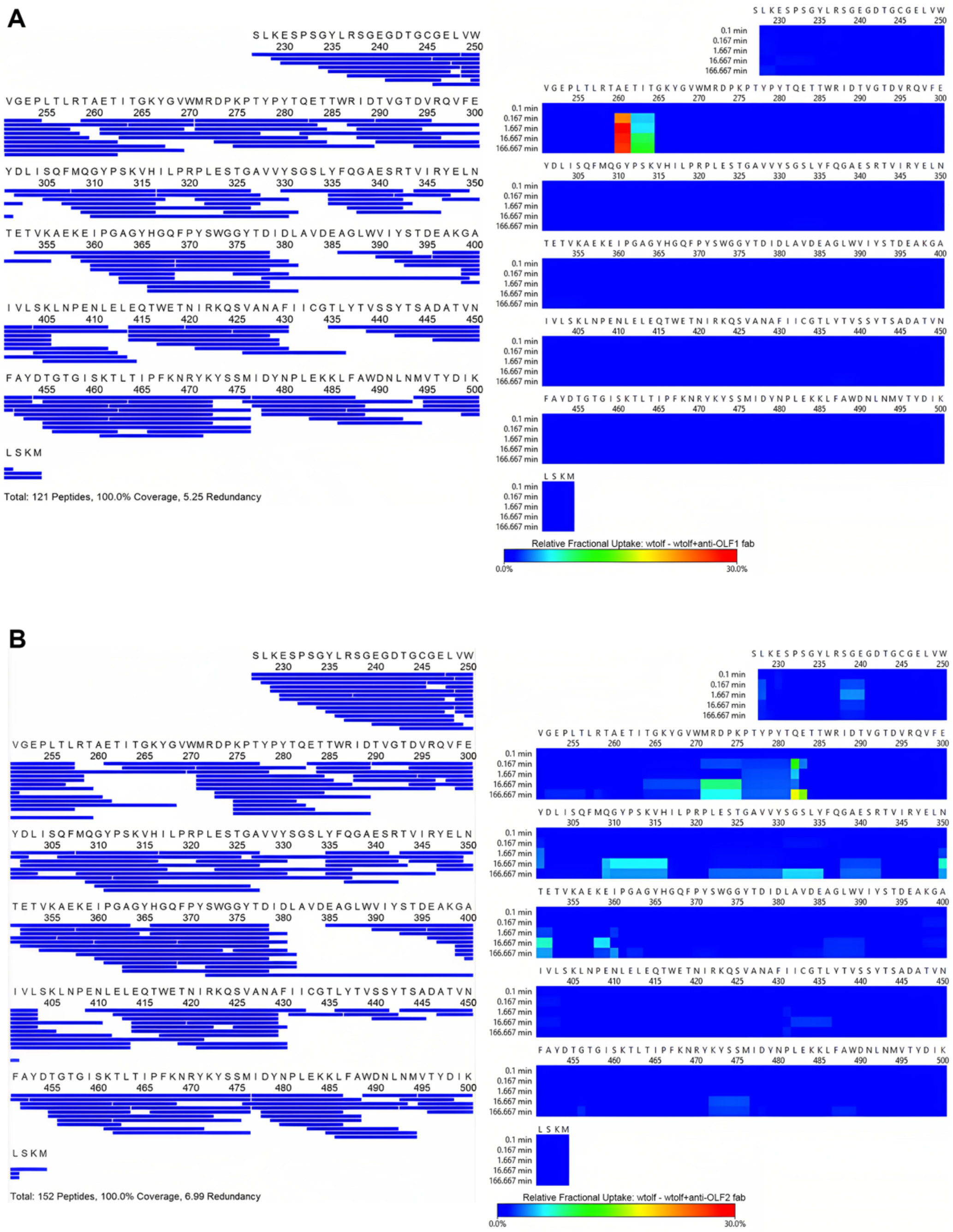

Fig. S5. HDX-MS data for unbound and antibody-bound WT OLF. Companion to Fig. 2E and F. For both A, anti-OLF1 Fab and B, anti-OLF2 Fab, coverage map (left) shows 100% HDX coverage for WT OLF sequence with each blue bar representing a peptic fragment reproducibly detected after HDX labeling reactions. Heat map (right) shows the difference in deuterium uptake between unbound and antibody-bound WT OLF for each labeling time point.

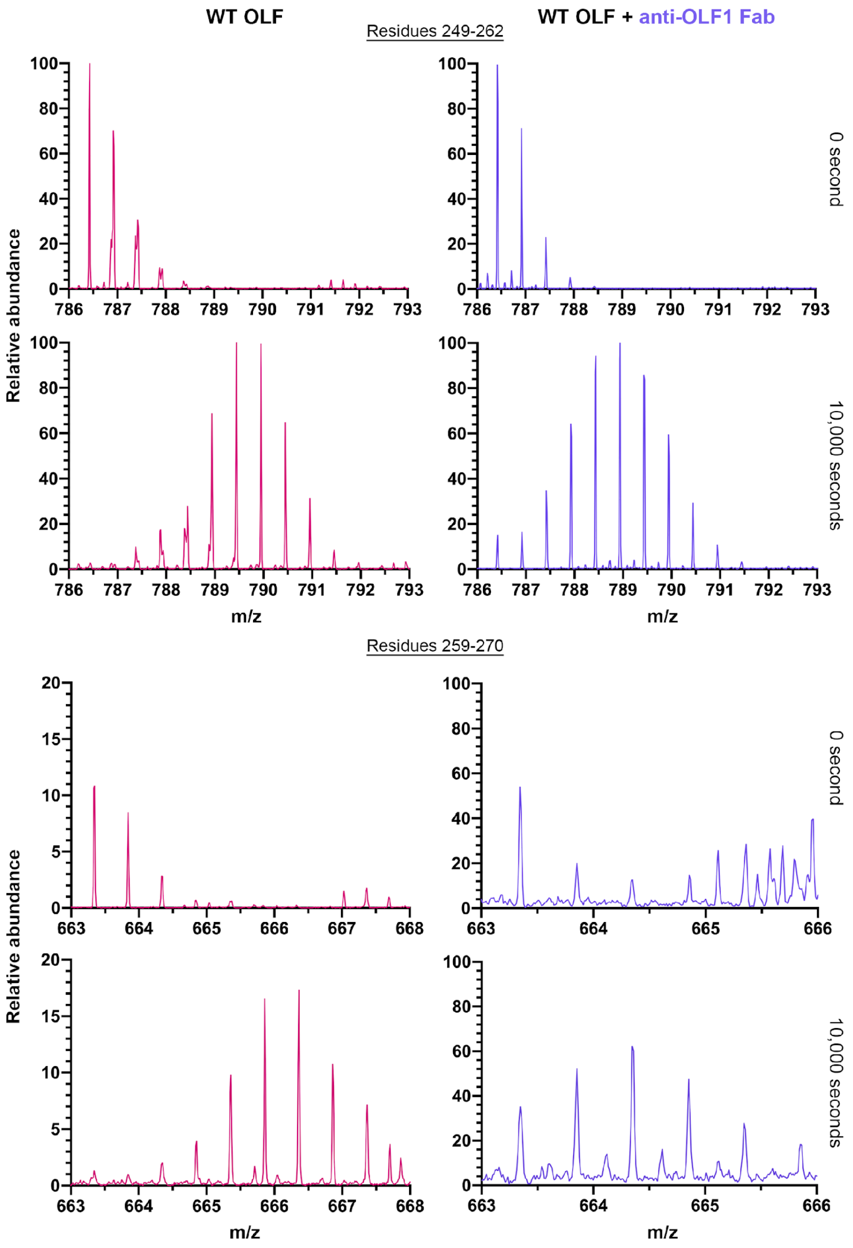

Fig. S6. Representative mass spectra of anti-OLF1 Fab binding epitope showed in Figure 2E after 10,000-second deuterium exchange.

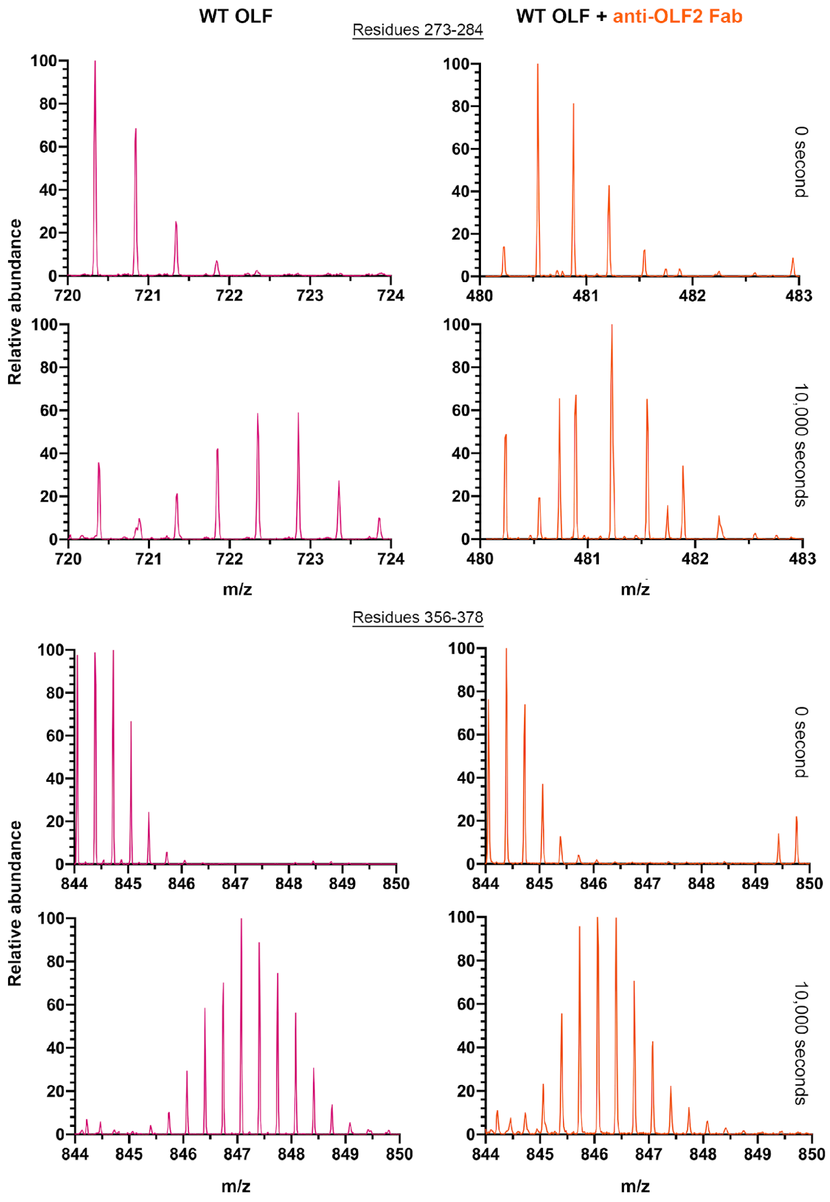

Fig. S7. Representative mass spectra of anti-OLF2 Fab binding epitope showed in Figure 2F after 10,000-second deuterium exchange.

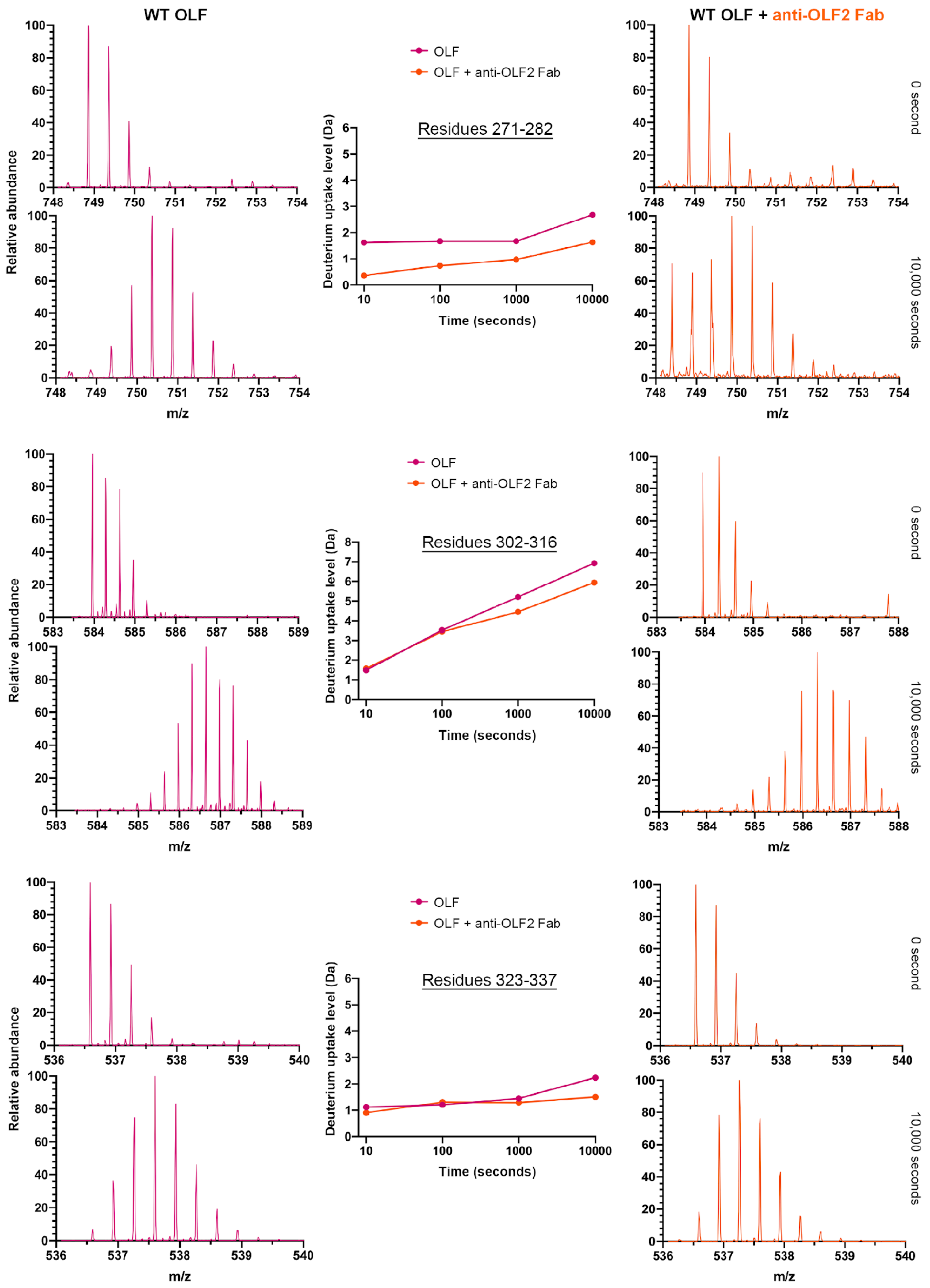

Fig. S8. Representative deuterium uptake graphs (middle) and mass spectra (left and right) of anti-OLF2 Fab binding epitope indicated in Figure 2F after 10,000-second deuterium exchange.

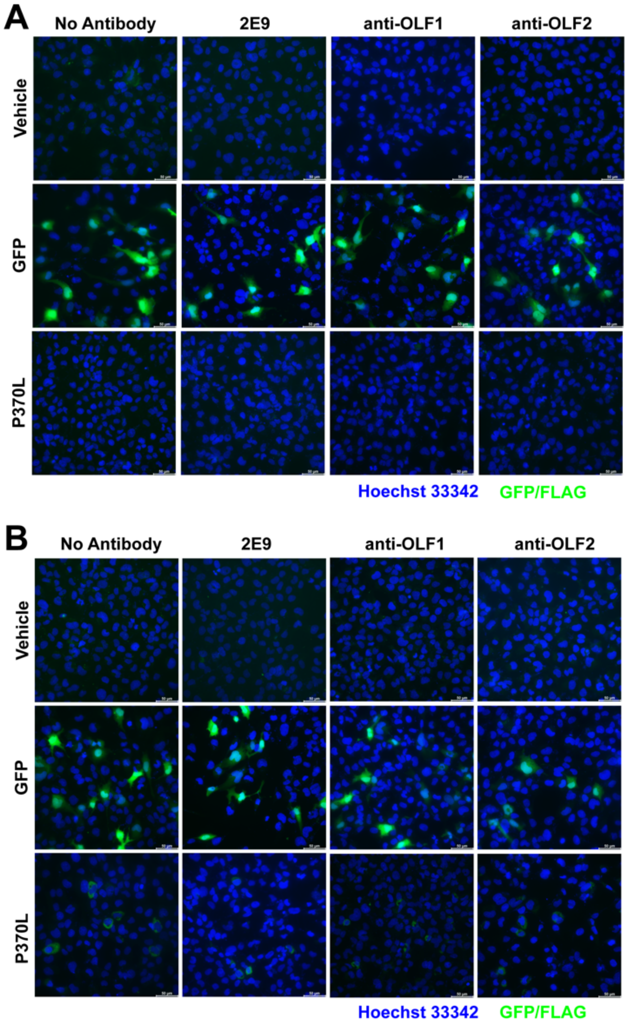

Fig. S9. Immunofluorescence imaging of HTM-1 cells. (**A**) Panels with only secondary antibodies (no primary). (**B**) Panels with primary and secondary antibodies. Myocilin^{P370L} panels are the same as in main Figure 3, top. Vehicle, lipofectamine only. Scale bar = 50 μm.

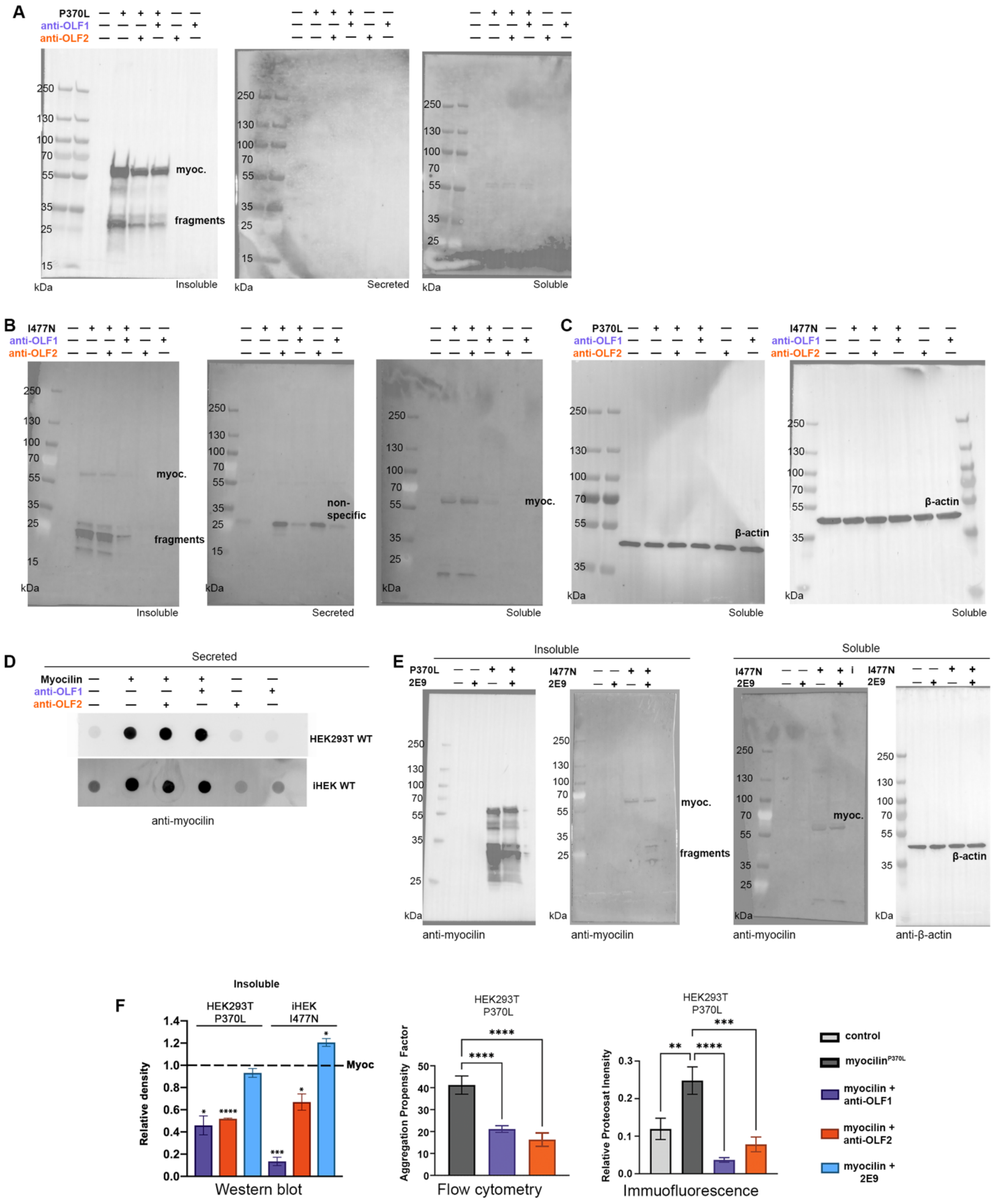
A
P370L
anti-OLF1
anti-OLF2
myoc.
fragments
kDa
Insoluble
Secreted
Soluble
B
I477N
non-specific
C
β-actin
D
Secreted
Myocilin
HEK293T WT
iHEK WT
anti-myocilin
E
2E9
Insoluble
Soluble
anti-β-actin
F
Insoluble
HEK293T P370L
iHEK I477N
Relative density
Myoc
Western blot
Aggregation Propensity Factor
Flow cytometry
Relative Proteosat Inensity
Immuofluorescence
control
myocilin
myocilin + anti-OLF1
myocilin + anti-OLF2
myocilin + 2E9

Fig. S10. Full immunoblots of secretion assays for HEK293T cell models and quantitation data. (**A**), Full Western blot analysis of myocilin^{P370L} levels in the insoluble, secreted, and soluble fractions obtained from HEK293T cells. (**B**), Full Western blot analysis of myocilin^{I477N} levels in the insoluble, secreted, and soluble fractions obtained from iHEK^{I477N} cells. **C**, Western blot analysis of β-actin levels in the same soluble fractions in **A** and **B** as a loading control. (**C**), Dot blot analysis of WT myocilin levels in HEK293T (top) and iHEK^{WT} (bottom) cells. Transfection with either anti-OLF1 or anti-OLF2 does not alter WT myocilin secretion in either cell lines. (**D**) Dot blot of secreted WT myocilin co-coexpressed alone or with anti-OLF1 or anti-OLF2 in HEK293T (top) and iHEK^{WT} (bottom). (**E**), Full Western blot analysis of transfection with the negative control antibody 2E9. Data in A-E represent at least two biological replicates. (**F**), Quantitation of results presented in panels A, B, and E as well as Figure 4. Quantification of Western blots of insoluble myocilin^{P370L} and myocilin^{I477N} is the mean±SD of two biological replicates and normalized to myocilin level in the absence of antibodies. Statistical significance for Westerns was assessed with unpaired two-tailed *t*-tests. Quantification of flow cytometry data to measure the aggregation propensity factor was conducted on two biological replicates each with three technical replicates. Quantification of immunofluorescence was performed with relative intensity of PROTEOSTAT normalized to calnexin with Image J and includes two biological replicates each with two technical replicates (right). Statistical significance was accessed with One-way analysis of variance (ANOVA) with Dunnett's multiple comparisons test for post-analysis. Ns, not significant; *P<0.05; **P<0.01; ***P<0.001; ****P<0.0001. See Table 2 for list of commercial antibodies used for immunoblotting.

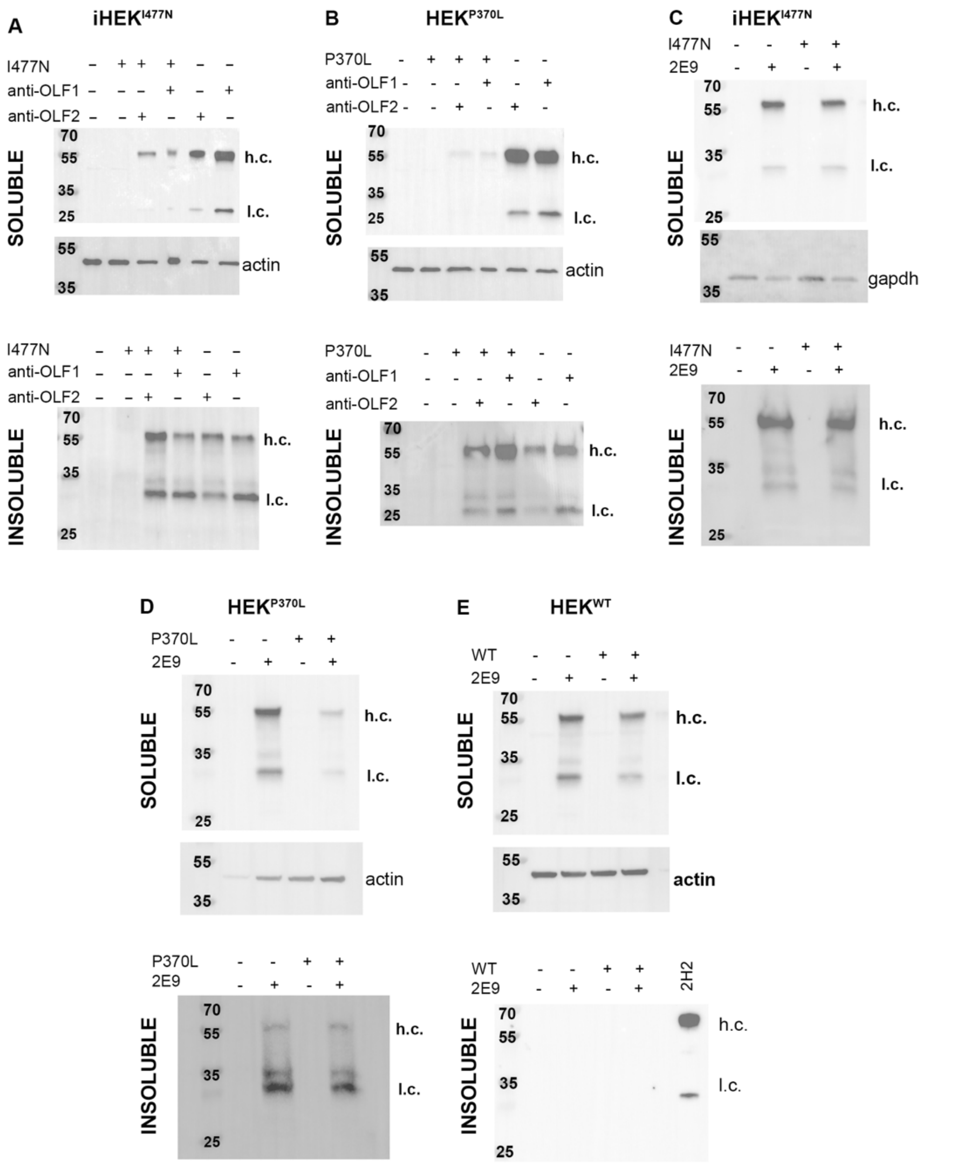

Fig. S11. Tracking of anti-OLF1, anti-OLF2, and 2E9 across cellular experiments. Western blots of soluble and insoluble fractions for (**A**) iHEK^{I477N} for experiments involving anti-OLF1 and anti-OLF2. (**B**) HEK^{P370L} for experiments involving anti-OLF1 and anti-OLF2. (**C**)iHEK^{I477N} for experiments involving 2E9. (**D**) HEK^{P370L} for experiments involving 2E9. (**E**) Soluble and insoluble fractions for HEK^{WT} for experiments involving 2E9. h.c., antibody heavy chain; antibody l.c. light chain. 2H2 is a custom antibody with an epitope to the N-terminal region of myocilin (4).

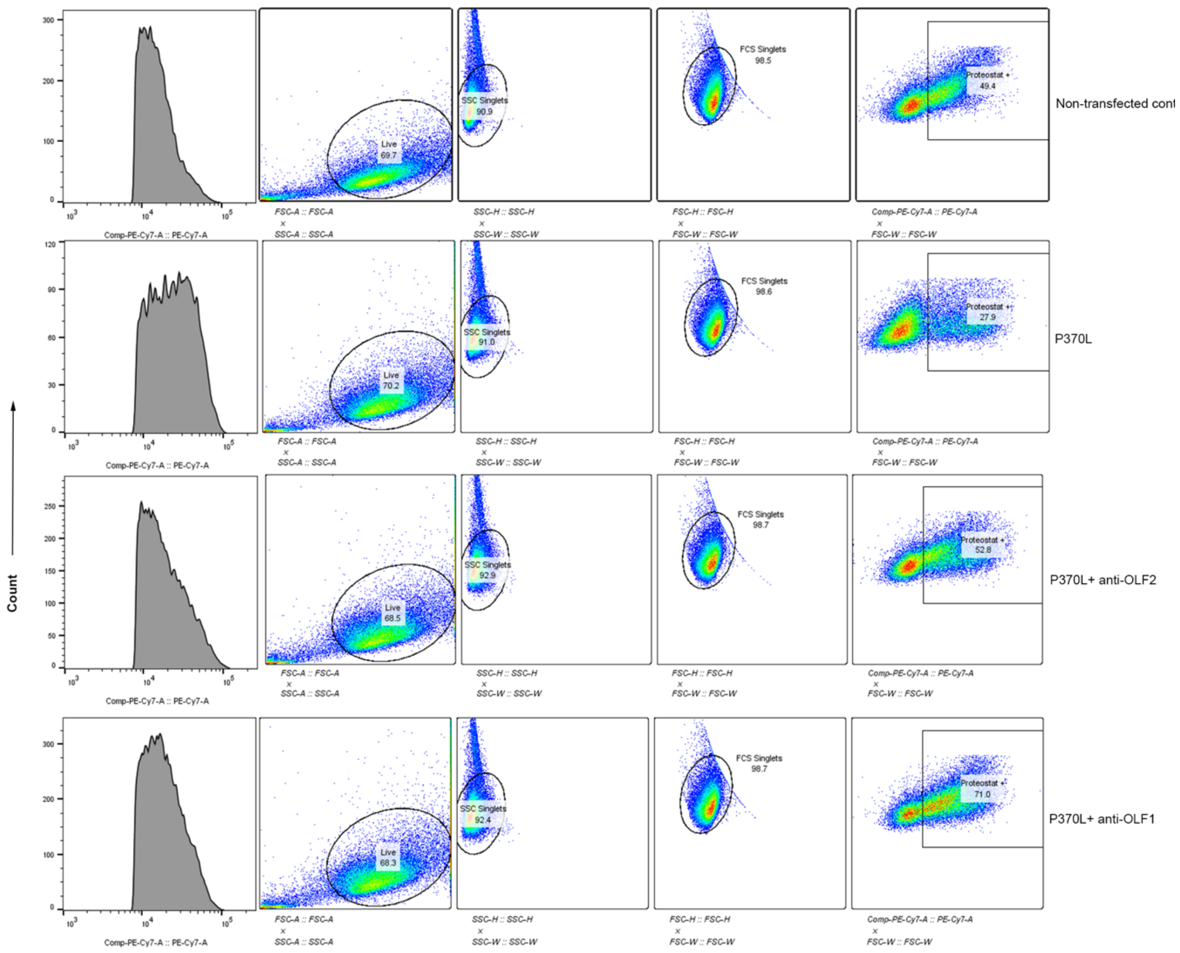

Fig. S12. Raw data used in flow cytometry analysis. Cells (50,000) were collected for each technical replicate and gated in the scheme above. Cells were gated first according to size using a forward scatter (FSC-A) versus side scatter (SSC-A) plot to determine live versus dead cells. Cells were then gated to exclude doublets and cell clumps using double discrimination of Scatter (SSC-H versus SSC-W) and SSC singlets (FSC-H versus FSC-W). PROTEOSTAT positive cell population was represented as a histogram.

A

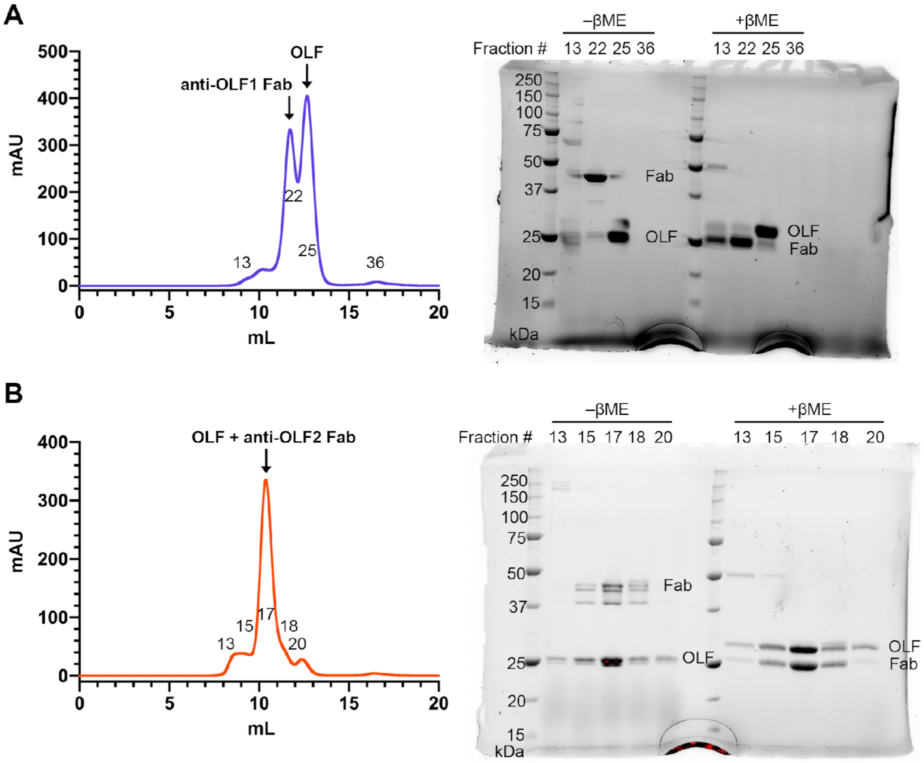

B

Fig. S13. Size-exclusion chromatography and fractional SDS-PAGE analysis of WT OLF complexed with A, anti-OLF1 Fab and B, anti-OLF2 Fab. SEC fraction numbers are indicated on each chromatogram and the corresponding lanes on each gel.

Supporting References

1. S. E. Hill, R. K. Donegan, R. L. Lieberman, The glaucoma-associated olfactomedin domain of myocilin forms polymorphic fibrils that are constrained by partial unfolding and peptide sequence. *J Mol Biol* **426**, 921-935 (2014).
2. H. F. Scelsi, K. R. Hill, B. M. Barlow, M. D. Martin, R. L. Lieberman, Quantitative differentiation of benign and misfolded glaucoma-causing myocilin variants on the basis of protein thermal stability. *Dis Model Mech* **16** (2023).
3. J. E. Tropea, S. Cherry, D. S. Waugh, Expression and purification of soluble His(6)-tagged TEV protease. *Methods Mol Biol* **498**, 297-307 (2009).
4. A. C. Patterson-Orazem *et al.*, Recombinant antibodies recognize conformation-dependent epitopes of the leucine zipper of misfolding-prone myocilin. *J Biol Chem* **297**, 101067 (2021).
5. D. J. E. Huard, A. P. Jonke, M. P. Torres, R. L. Lieberman, Different Grp94 components interact transiently with the myocilin olfactomedin domain in vitro to enhance or retard its amyloid aggregation. *Sci Rep* **9**, 12769 (2019).
